# Synthesis, Structural Characterization, and Application of a Cationic Thermo-Responsive Copolymer Based on [2-(Methacryloyloxy)ethyl]trimethylammonium Chloride and *N*-Vinylcaprolactam for Use with Butyl Xanthate in the Flotation of Ultrafine Chalcopyrite

**DOI:** 10.3390/polym18212654

**Published:** 2026-10-26

**Authors:** Nazira Samenova, Nessipbay Tussupbayev, Bakyt Daulet, Sabira Narbekova, Zarina Kenessova

**Affiliations:** 1 Institute of Metallurgy and Ore Beneficiation JSC, Satbayev University, Almaty 050010, Kazakhstan; nazira.orakkyzy@gmail.com (N.S.); b.d.e.metal@mail.ru (B.D.); s.narbekova@mail.ru (S.N.); 2 Department of Chemistry and Technology of Organic Substances, Natural Compounds and Polymers, Faculty of Chemistry and Chemical Technology, Al-Farabi Kazakh National University, Almaty 050040, Kazakhstan; zarina.kenesova@gmail.com

**Keywords:** cationic copolymer, thermo-responsive polymer, METAC, *N*-vinylcaprolactam, RAFT polymerization, ultrafine chalcopyrite, flotation, interfacial interaction, XPS

## Abstract

The flotation of finely divided chalcopyrite and quartz was investigated using a combined reagent system based on butyl xanthate and a thermosensitive cationic copolymer. Copolymers of [2-(methacryloyloxy)ethyl]trimethylammonium chloride and *N*-vinylcaprolactam (co(METAC/NVCL)), prepared by RAFT polymerisation, were used as the polymer components. Their structure and physicochemical properties were investigated using IR and NMR spectroscopy, gel permeation chromatography and thermal analysis. Flotation tests were carried out on an artificial mixture of chalcopyrite and quartz in a mass ratio of 1:1 at pH 8.5 and temperatures of 20–50 °C. At 40 °C, the combined use of a 70:30 copolymer and butyl xanthate, with each component at a concentration of 5 mg/L, resulted in a copper recovery of 77.68% when the copper content in the concentrate was 30.90%, and a SiO_2_ recovery of 5.78%. Compared with the xanthogenate process, copper recovery increased by 5.62%, whilst SiO_2_ recovery decreased by 1.10%.

## 1. Introduction

The development of functional polymer materials capable of altering their physicochemical properties in response to external factors is a rapidly advancing area of modern polymer science. Stimuli-responsive polymers whose properties can be modulated by changes in temperature, pH, ionic strength, and medium composition are of particular interest. The ability to precisely tailor the chemical composition and macromolecular architecture allows these materials to combine adsorption, interfacial, flocculation, and surface-active properties. This opens up prospects for their application in separation processes requiring controlled interaction between the polymer and a dispersed solid phase [1,2]. A recent review of polymeric collectors indicates that polymer-based reagents including nanoparticles, thermosensitive systems, and block copolymers can achieve recovery rates and concentrate quality comparable to, or in some cases superior to, those obtained with traditional low-molecular-weight collectors.

One of the most challenging areas of application for such reagents is the flotation recovery of fine and ultrafine mineral particles. As particle size decreases, the probability of effective collision and attachment to air bubbles diminishes, while the influence of slime coatings, surface charge, and interparticle interactions increase.

Flotation processing of chalcopyrite (CuFeS_2_), one of the main copper sulphide minerals, is widely used in the processing of copper-bearing ores. Despite the relatively high natural floatability of chalcopyrite under certain conditions and the effectiveness of traditional xanthate collectors, the recovery of finely divided particles remains a significant technical challenge. As particle size decreases, the likelihood of effective collision and attachment to air bubbles diminishes, the effect of surface oxidation intensifies, and the risk of mechanical carry-over of gangue minerals into the concentrate increases. Consequently, current research is focused not only on enhancing the collecting ability of reagents, but also on ensuring selectivity, regulating interphase interactions and improving the flotation characteristics of finely dispersed mineral systems.

The efficiency of the flotation separation of chalcopyrite is largely determined by the physico-chemical state of the mineral surface, its hydration and the nature of its interaction with flotation reagents. A review [3] of electrochemical studies of sulphide minerals examined the characteristics of redox processes on the surfaces of pyrite, chalcopyrite and galena, as well as the influence of electrochemical interactions on the behaviour of minerals in flotation systems. In [4], using the self-consistent charge method in the strong-coupling density functional theory (SCC-DFTB) approximation, a computational approach was developed to investigate interphase interactions in systems comprising chalcopyrite and pyrite. The authors emphasised the importance of taking into account surface hydration, temperature and the adsorption of flotation reagents in order to provide a more complete description of the processes occurring at the mineral–solution interface.

Significant difficulties arise in the selective flotation of chalcopyrite in the presence of pyrite, which has similar surface and electrochemical characteristics. In [5], the effect of pyrite content and regrinding conditions on the flotation performance of chalcopyrite was investigated. It was found that changes in the composition of the grinding media and the amount of pyrite influence the formation of hydrophilic iron-containing compounds on the mineral surface and, consequently, the efficiency of sulphide separation. These results show that flotation selectivity is determined not only by the chemical nature of the collector, but also by the condition of the mineral surface, which is formed during pulp preparation and processing.

One of the current approaches to improving selectivity is the development of collectors with functional groups that ensure preferential interaction with the surface of the target mineral. Thus, in [6], tetramethylthiourea (TMTU) was investigated as a selective collector for chalcopyrite during its separation from pyrite under weakly alkaline conditions. At a TMTU concentration of 2 × 10^−5^ mol/L, the recovery of chalcopyrite reached approximately 85 per cent, whilst the recovery of pyrite remained below 10 per cent. The results of surface studies and quantum-chemical calculations confirmed the preferential interaction of TMTU with chalcopyrite.

Alongside the development of low-molecular-weight collectors, the use of functional polymers capable of altering the surface and aggregation properties of finely dispersed particles is of considerable interest. In [7], the use of polyvinyl acetate (PVAc) as a selective flocculant in the reverse flotation of talc from a mixture with chalcopyrite was investigated. It was found that PVAc promotes the preferential aggregation of finely dispersed talc particles due to specific intermolecular interactions, thereby enabling their separation from chalcopyrite. This study demonstrates that the use of polymeric reagents can be directed not only towards altering the hydrophobicity of minerals, but also towards regulating their selective aggregation.

Polymeric reagents are of particular interest for addressing this issue, as they can act not only as wettability modifiers but also as flocculants or aggregating agents, thereby increasing the effective size of ultrafine particles and enhancing the likelihood of their interaction with bubbles [8,9]. For thermo-responsive polymers, an additional advantage is the ability to reversibly alter hydration, conformation, and interparticle interactions by changing the temperature.

Poly(*N*-vinylcaprolactam) (PNVCL) is a prominent example of a thermosensitive polymer. In aqueous solutions, PNVCL exhibits LCST-type, temperature-dependent behavior driven by the changing balance between polymer–water and polymer–polymer interactions. Below the phase transition temperature, the macromolecular chain exists primarily in a hydrated state, whereas increasing the temperature leads to reduced hydration and a transition to a more compact chain conformation. For PNVCL, the transition temperature depends on molecular weight, macromolecular architecture, copolymer composition, and solution conditions [10]. These characteristics make NVCL a promising monomer for the synthesis of functional polymers with tunable interfacial properties.

The controlled polymerization of NVCL is of particular interest because it enables the precise control of the molecular weight characteristics and structure of the resulting materials. The reversible addition–fragmentation chain-transfer (RAFT) method has previously been successfully employed for the synthesis of PNVCL and statistical NVCL copolymers. The RAFT approach allows for the production of NVCL-containing copolymers with controlled dispersity, while varying the nature and content of the comonomer leads to a significant change in the cloud point temperature of the polymer system [11]. This confirms the feasibility of tuning the thermo-responsive behavior of NVCL-containing macromolecules by modifying their chemical composition.

Alongside thermosensitivity, charge density is a key parameter for a functional polymer intended to interact with a mineral surface. Cationic polymers can interact with negatively charged surface sites via electrostatic attraction. For instance, studies on the use of polyether amines as quartz collectors have shown that their adsorption is primarily governed by electrostatic interactions between protonated amino groups and the negatively charged mineral surface, with a supplementary contribution from hydrogen bonding [12,13].

In this regard, [2-(methacryloyloxy)ethyl]trimethylammonium chloride (METAC) is of particular interest as a monomer containing a quaternary ammonium group. This group imparts a permanent positive charge to the polymer chain and allows for the modulation of electrostatic interactions with negatively charged surfaces. METAC has already been used to prepare cationic thermo-responsive polymer systems, where varying the cationic monomer content significantly influenced hydration, phase behavior, and intermolecular interactions [14]. The cationic nature of METAC is directly attributable to the presence of the quaternary ammonium center.

Studies of copolymers of *N*-vinylcaprolactam with *N*,*N*-dimethylaminoethyl methacrylate are particularly relevant to the system under consideration. Shatalov et al. synthesized such thermosensitive copolymers and demonstrated that the presence of a nitrogen-containing methacrylate unit imparts polyelectrolyte and flocculating properties to them [10]. These results demonstrate the feasibility of combining NVCL-based thermosensitive segments and cationic functional groups within a single macromolecule. However, the METAC used in the present work differs fundamentally in that it contains a permanently charged quaternary ammonium group rather than a pH-dependent tertiary amino group.

A number of studies confirm the potential of cationic polymer systems for the flotation of sulfide minerals. Research into the use of cationic polystyrene nanoparticles as collectors for chalcopyrite flotation has demonstrated that their efficiency can be comparable to that of traditional collectors. The authors attributed the attachment of the particles to the chalcopyrite surface to electrostatic interactions, while the incorporation of imidazole functional groups further enhanced the affinity for surface copper sites [15,16,17].

Studies investigating PNVCL specifically within flotation processes are of particular interest, such as the application of polyvinylcaprolactam in reagent regimes for the flotation of gold-bearing minerals [18]. Subsequent research examined the interaction of modified PNVCL with chalcopyrite, galena, and pyrite. It was shown that the modified polymer adsorbs onto the surface of sulfide minerals and alters their flotation behavior; specifically, an increase in the floatability of chalcopyrite and galena was observed, with a significantly lesser effect on pyrite [19].

Further confirmation of the functional role of PNVCL was obtained during the flotation of finely dispersed gold-bearing products. Modified PNVCL promoted the selective aggregation of fine particles and, when used in conjunction with a xanthate collector, resulted in increased gold recovery. These results indicate that the mechanism of action of NVCL-containing polymers may involve not only the alteration of surface hydrophobicity but also the controlled aggregation of fine mineral particles [20,21].

Despite advances in polymer-based flotation reagents, the performance of cationic NVCL-containing copolymers as additives in xanthate-based systems for ultrafine mineral separation requires further clarification. In particular, it is important to examine how variations in the METAC/NVCL feed ratio, accompanied by changes in molar mass, relate to interfacial behaviour and flotation performance. Comparative tests on chalcopyrite–quartz mixtures can help assess the contribution of these copolymers to the separation achieved using a conventional xanthate collector.

In this study, cationic co(METAC/NVCL) copolymers were synthesized via RAFT polymerization at initial METAC/NVCL molar feed ratios of 90:10, 80:20, and 70:30. Their structure and physicochemical properties were investigated using FTIR, ^1^H and ^13^C NMR spectroscopy, gel permeation chromatography, and thermal analysis. The effects of copolymer formulation and dosage on the flotation recovery of ultrafine chalcopyrite (<30 μm) were examined. The 70:30 formulation was further evaluated in combination with butyl xanthate for the flotation separation of a synthetic chalcopyrite–quartz mixture at pH 8.5 and temperatures of 20–50 °C. The combined reagent system was compared with butyl xanthate alone to assess the effect of adding the copolymer on copper recovery, concentrate grade, and SiO_2_ recovery. Contact-angle measurements and XPS analysis provided complementary information on interfacial behaviour and the surface composition of treated chalcopyrite. The study aimed to characterize the copolymers and evaluate their potential as additives for improving chalcopyrite–quartz separation under the conditions investigated.

## 2. Materials and Methods

### 2.1. Materials

[2-(Methacryloyloxy)ethyl]trimethylammonium chloride (METAC), *N*-vinylcaprolactam (NVCL), 4-cyano-4-(phenylcarbonothioylthio)pentanoic acid as a chain transfer agent, and 2,2′-azobis(2-methylpropionamidine) dihydrochloride as a radical initiator were used for the synthesis of the copolymers. All reagents were purchased from Sigma-Aldrich (St. Louis, MO, USA) and used without further purification.

### 2.2. Synthesis of METAC–NVCL Copolymers via RAFT Polymerization

METAC and NVCL were charged into a 50 mL three-necked glass flask equipped with a mechanical stirrer, a reflux condenser, and an inert gas (nitrogen) supply system, using molar ratios of 90:10, 80:20, and 70:30, respectively.

**Figure 1:**
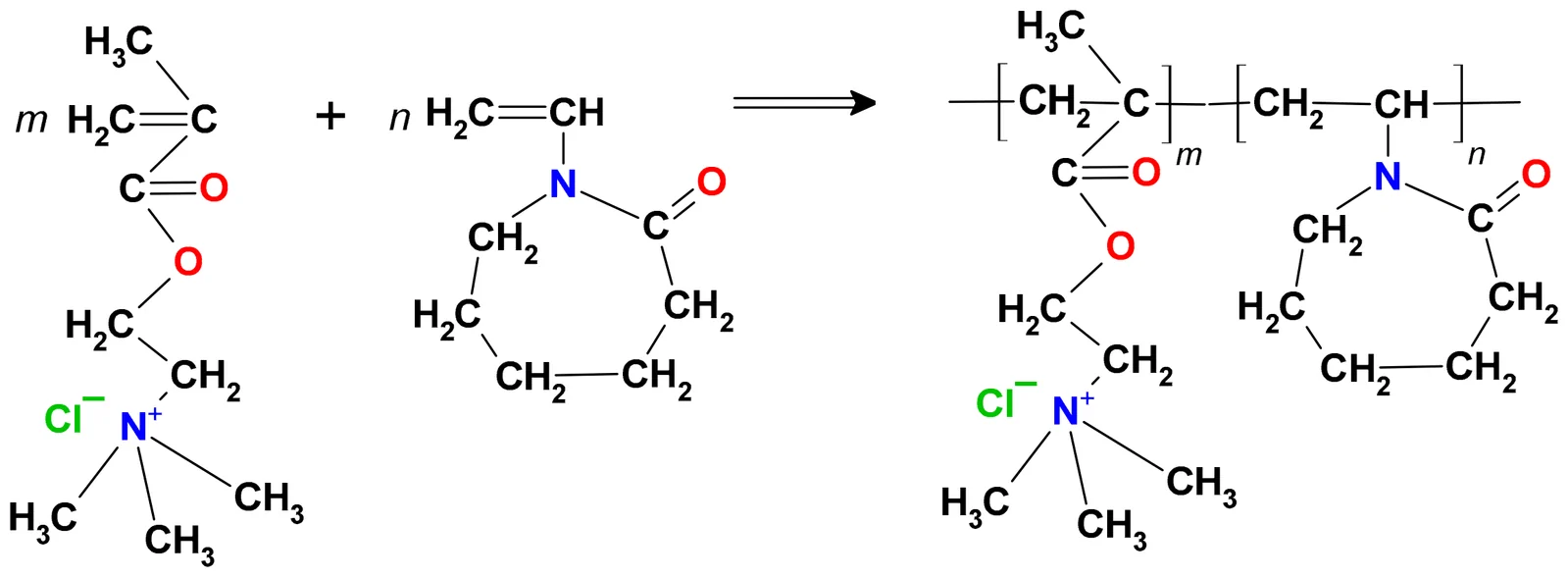
Scheme of the copolymerization of METAC and *N*-vinylcaprolactam (NVCL) to form the co(METAC/NVCL) copolymer.

The RAFT agent 4-cyano-4-(thiobenzoylthio)pentanoic acid (CTBPA) and the initiator 2,2′-azobisisobutyronitrile (AIBN) were added to the reaction mixture (Figure 1). The initiator content was 0.05 wt% of the total monomer mass, with an initial molar ratio ([CTBPA]_0_/[AIBN]_0_) of 14.

A 1:1 (by mass) mixture of water and ethanol was used as the solvent. The total monomer concentration in the reaction mixture was 50 vol%. The mixture was stirred until a homogeneous solution was obtained, followed by degassing via nitrogen purging for 5 min to remove dissolved oxygen.

Polymerization was carried out at 75 °C for 24 h under constant stirring. Upon completion of the reaction, the mixture was cooled to room temperature. The resulting copolymer was precipitated in diethyl ether, separated from the liquid phase, and redissolved in water. The aqueous copolymer solution was then precipitated again in diethyl ether. The reprecipitation procedure was repeated three times to remove unreacted monomers and low-molecular-weight impurities.

The purified copolymers were dried in a vacuum oven at 25 °C to constant mass. The yield of the METAC–NVCL copolymer was 79.2 wt% for the 90:10 composition, 75.9 wt% for the 80:20 composition, and 72.7 wt% for the 70:30 composition.

### 2.3. Methods for Copolymer Investigation

#### 2.3.1. NMR Spectroscopy

**Figure A2:**
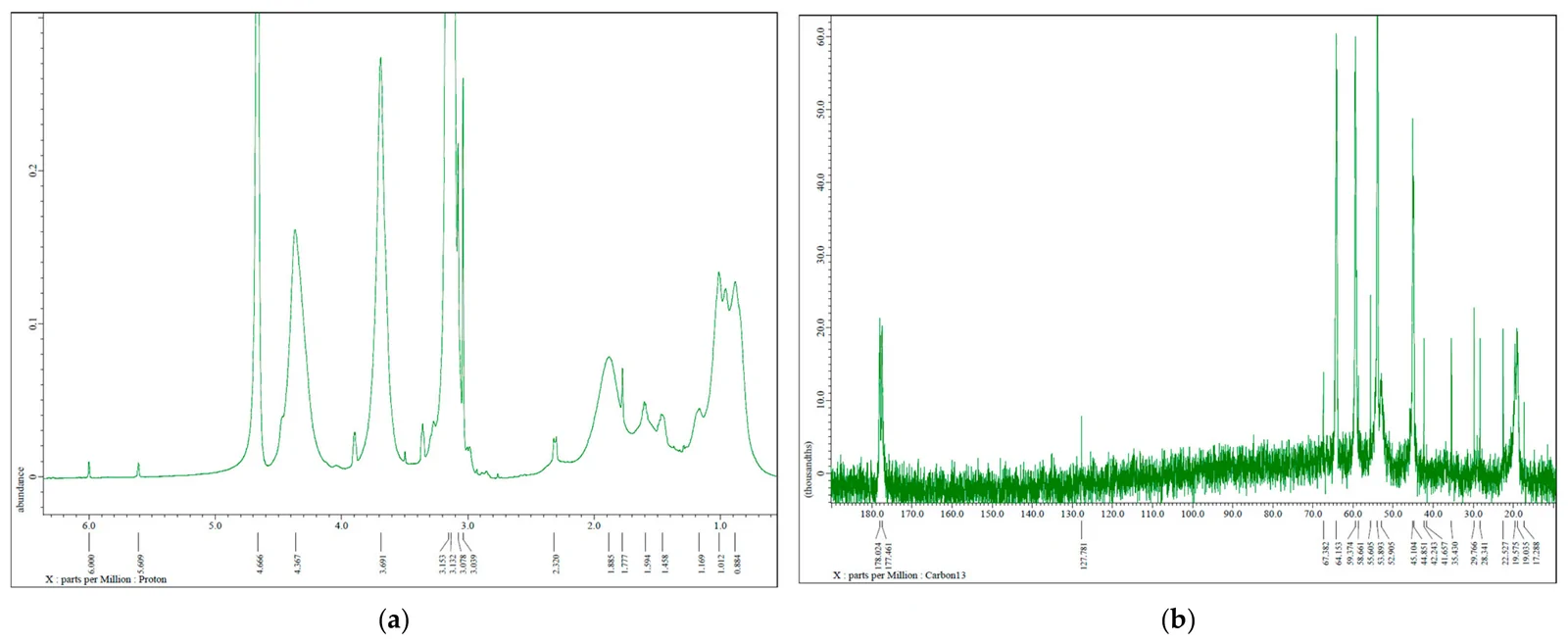
(**a**) ^1^H and (**b**) ^13^C NMR spectra of the co(METAC/NVCL) copolymer with a 90:10 molar ratio.

**Figure A3:**
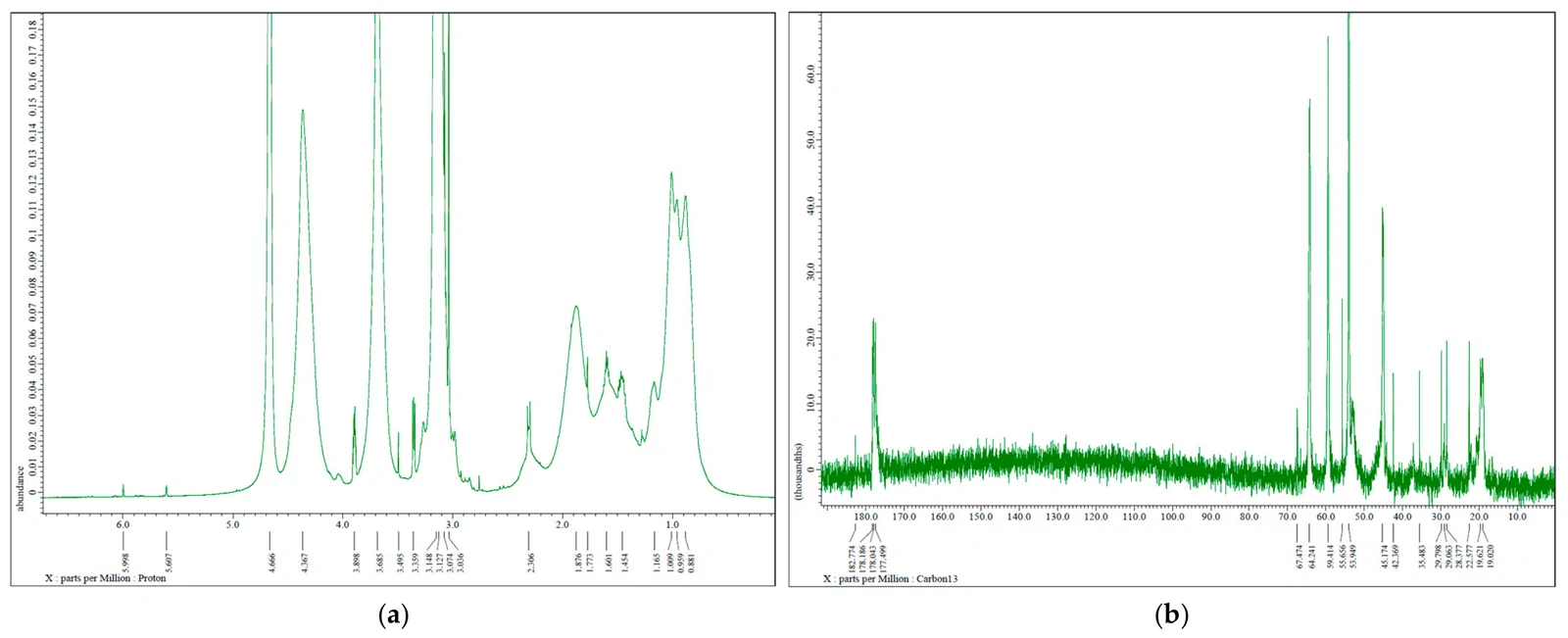
(**a**) ^1^H and (**b**) ^13^C NMR spectra of the co(METAC/NVCL) copolymer with an 80:20 molar ratio.

**Figure A4:**
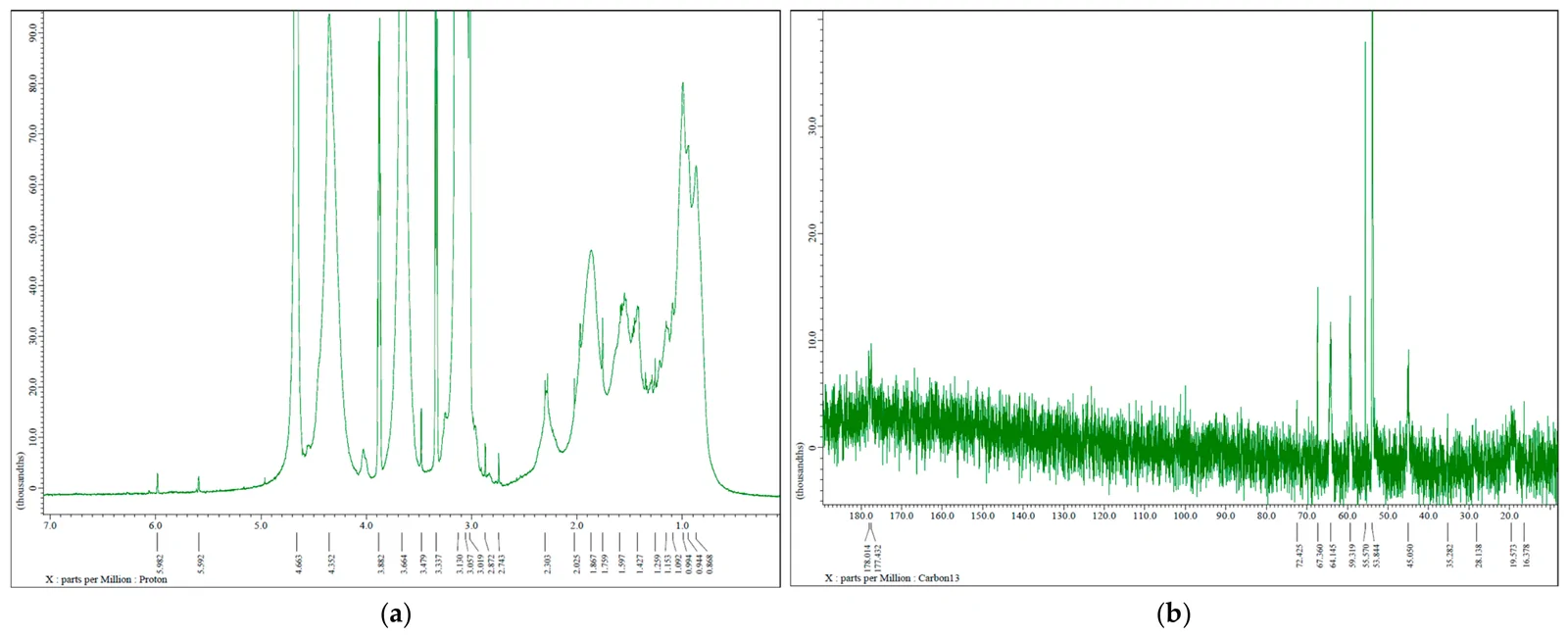
(**a**) ^1^H and (**b**) ^13^C NMR spectra of the co(METAC/NVCL) copolymer with a 70:30 molar ratio.

The structure of the synthesized copolymers of [2-(methacryloyloxy)ethyl]trimethylammonium chloride (METAC) and *N*-vinylcaprolactam (NVCL) was investigated using ^1^H and ^13^C NMR spectroscopy. Measurements were performed on a JNM-ECA 500 NMR spectrometer (JEOL, Tokyo, Japan). Samples were dissolved in deuterated water (D_2_O) and analyzed at room temperature in standard 5 mm NMR tubes. Sixty-four scans were used to record the ^1^H NMR spectra. Following the acquisition of the free induction decay (FID) signal, Fourier transformation, phase correction, and baseline correction were performed. Three copolymer samples, obtained with initial METAC/NVCL molar ratios of 90:10, 80:20, and 70:30, were analyzed. The full ^1^H and ^13^C NMR spectra are presented in Appendix A (Figure A2, Figure A3 and Figure A4 and Table A1, Table A2 and Table A3).

#### 2.3.2. IR Spectroscopy

The IR spectra of the starting monomers and synthesized copolymers were recorded using a Nicolet iS10 Fourier-transform infrared (FTIR) spectrometer Thermo Scientific (Waltham, MA, USA) equipped with an attenuated total reflection (ATR) accessory. The samples were analyzed directly, without prior KBr pellet preparation. Spectra were recorded in the wavenumber range of 4000–400 cm^−1^. The obtained spectra were analyzed based on the position and intensity of characteristic absorption bands to confirm the presence of METAC and NVCL functional groups in the structure of the synthesized copolymers.

#### 2.3.3. TGA and DSC

The thermal properties of the copolymers were investigated using an STA 6000 simultaneous thermal analyzer PerkinElmer (Shelton, CT, USA). The sample mass was 33.105 mg. The analysis was conducted in a nitrogen atmosphere (cell volume 30 mL, flow mode) under the following conditions: initial temperature of 30 °C; isothermal hold for 1 min at 30 °C; and heating from 30 to 800 °C at a rate of 10 °C/min. The instrument was calibrated prior to measurements. Temperature calibration was performed using indium (Tm = 156.6 °C) and silver (Tm = 960.8 °C) standards; the measured values were 157.6 °C and 961.7 °C, respectively. Indium was used for heat flow calibration (enthalpy of fusion 28.45 J/g; measured value 22.1 J/g for a mass of 23.153 mg). The calculated low-range coefficient was 1.287, confirming the correct instrument setup.

### 2.4. Flotation

Single-mineral flotation experiments were conducted using an FML-0.1 laboratory flotation machine JSC RPC Mekhanobr-Tekhnika (Saint Petersburg, Russia). Each test used a 5.0 g chalcopyrite sample with a particle size of −30 + 0 μm and a mean particle diameter of 18.6 μm. The reagent system comprised butyl xanthate and a cationic co(METAC/NVCL) copolymer prepared at an initial METAC/NVCL feed ratio of 90:10, 80:20, or 70:30. Copolymer stock solutions were prepared in deionized water at a concentration of 0.5 wt%. The final copolymer concentrations in the flotation suspension were 2.0, 5.0, and 7.5 mg/L, while butyl xanthate was used at concentrations of 2.0, 5.0, and 7.5 mg/L. Before flotation, the suspension pH was adjusted to 8.5 ± 0.1 using NaOH solution, and the temperature was maintained at 40 ± 1.0 °C. Once the target temperature was reached, the required amount of copolymer solution was added to the suspension. The conditioning time following copolymer addition was 3 min. Flotation was subsequently performed for 5 min under constant agitation and aeration conditions.

To evaluate the effect of temperature on chalcopyrite–quartz separation using the combined reagent system, additional flotation experiments were conducted on a synthetic mixture containing chalcopyrite and quartz in a mass ratio of 1:1. The total sample mass was 10.0 g. Tests were performed at pH 8.5 and temperatures of 20, 30, 40, and 50 °C. The co(METAC/NVCL) copolymer prepared at an initial METAC/NVCL feed ratio of 70:30 was used in this series. The copolymer and butyl xanthate solutions were pre-mixed before addition to the flotation pulp, with a final concentration of 5 mg/L for each component. T-80 was added separately as a frother at a concentration of 15 mg/L. Reference tests were conducted using butyl xanthate alone at 5 mg/L, with the same frother concentration and otherwise comparable experimental conditions. Separation performance was evaluated from the concentrate mass yield, the Cu and SiO_2_ contents of the flotation products, and the corresponding recoveries.

### 2.5. Determination of the Contact Angle

Contact angle measurements on mineral surfaces were performed using an LK-1 goniometer. A pre-polished surface of a monomineralic chalcopyrite sample was used to investigate wettability. Prior to measurement, the chalcopyrite surface was conditioned with a xanthate solution at a concentration of 5 mg/L. Following treatment, a droplet of either distilled water (control) or a co(METAC/NVCL) copolymer solution (concentration 5 mg/L; METAC/NVCL ratios of 90:10, 80:20, and 70:30) was applied to the mineral surface. The contact angle was determined based on the droplet profile on the chalcopyrite surface.

### 2.6. X-Ray Photoelectron Spectroscopy of the Chalcopyrite Surface

X-ray photoelectron spectroscopy (XPS) was used to investigate the elemental composition and chemical state of the chalcopyrite surface before and after interaction with the cationic thermosensitive copolymer co(METAC/NVCL). Measurements were performed on a NEXSA spectrometer (Thermo Scientific, USA) using a monochromatic Al Kα source with an energy of 1486.6 eV. Spectra were recorded under high-vacuum conditions. For sample preparation, 2.0 g of chalcopyrite was dispersed in 100 mL of deionized water. The pH of the suspension was adjusted to a target value using a NaOH solution. To study the interaction between the copolymer and the mineral surface, a co(METAC/NVCL) solution with a concentration of 5 mg/L was added to the suspension. After 15 min of conditioning, the suspension was filtered; the solid phase was dried at room temperature and stored in a desiccator prior to XPS analysis. Untreated chalcopyrite served as the control sample.

Survey XPS spectra, as well as high-resolution spectra for the Cu 2p, Fe 2p, S 2p, O 1s, C 1s, and N 1s regions, were recorded for both the initial and co(METAC/NVCL)-treated chalcopyrite. Inclusion of the N 1s region allowed for the assessment of the presence of nitrogen-containing functional groups from the copolymer on the chalcopyrite surface. The binding energy scale was calibrated against the C 1s signal at 284.80 eV. Processing and deconvolution of high-resolution spectra were performed using OriginPro 2026 (version 10.35, OriginLab). A Shirley background was used for background correction. Individual spectral components were fitted with peak functions, and the quality of the fit was evaluated based on the agreement between the experimental and total calculated curves.

## 3. Results and Discussion

### 3.1. Characterization of Copolymers

**Figure 2:**
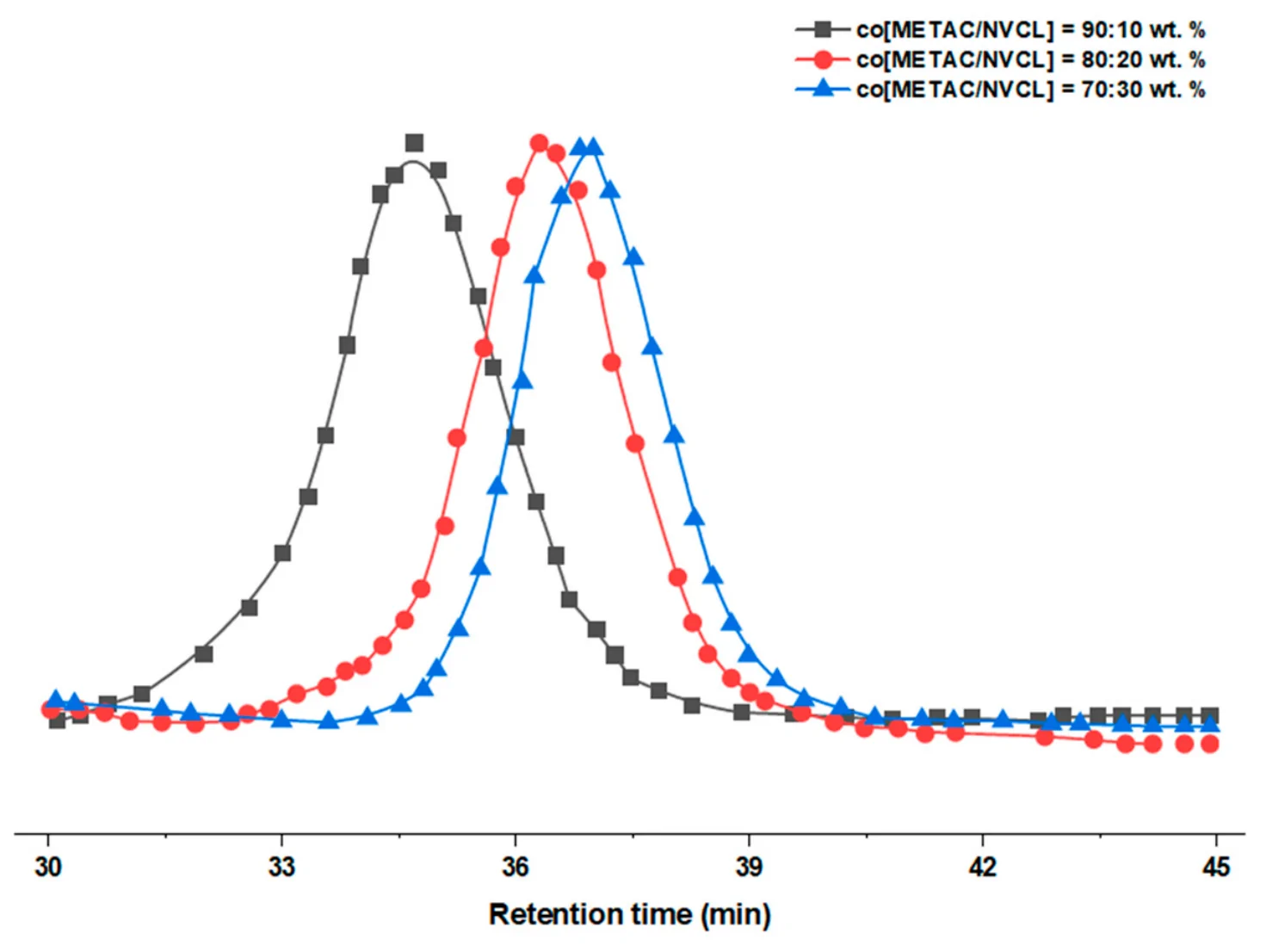
GPC chromatograms of METAC/NVCL copolymers with different mass ratios of monomer units (90:10, 80:20, and 70:30 wt%).

The molecular weight characteristics of the synthesized co(METAC/NVCL) copolymers were evaluated using gel permeation chromatography (GPC). Figure 2 shows the chromatograms of copolymers with different METAC/NVCL ratios, while the corresponding values for yield, number-average molecular weight (M_n_), weight-average molecular weight (M_w_), and dispersity (D) are listed in Table 1.

As shown in Figure 2, a change in copolymer composition is accompanied by a systematic shift in the chromatographic peak maximum toward longer retention times as the NVCL content increases. For the co(METAC/NVCL) 90:10 sample, the maximum appears at a shorter retention time, whereas for the 80:20 and 70:30 compositions, the peaks shift progressively to the right. This trend is consistent with the decrease in copolymer molecular weight as the NVCL fraction increases. The co(METAC/NVCL) 90:10 copolymer exhibited the highest molecular weight values: M_n_ = 11,150 g/mol and M_w_ = 16,230 g/mol. For the 80:20 composition, these values decreased to M_n_ = 9340 g/mol and M_w_ = 12,660 g/mol, while the lowest values—M_n_ = 6770 g/mol and M_w_ = 8180 g/mol—were obtained for co(METAC/NVCL) 70:30. Thus, increasing the NVCL content from 10% to 30% resulted in a progressive decrease in both number-average and weight-average molecular weights (Table 1).

#### 3.1.1. Structural Characterization of METAC–NVCL Copolymers by NMR

To confirm the chemical structure of the synthesized METAC–NVCL copolymers, the ^1^H and ^13^C NMR spectra of samples with initial component ratios of 90/10, 80/20, and 70/30 were analyzed. The obtained spectra exhibit characteristic signals corresponding to the functional groups of both comonomer units, consistent with the formation of METAC–NVCL copolymer systems.

In the ^1^H NMR spectra of all samples examined, an intense group of resonances is observed in the region of approximately δ 3.0–3.2 ppm. These signals are primarily attributed to the protons of the three methyl groups of the quaternary ammonium moiety, N^+^(CH_3_)_3_, which is characteristic of METAC units. The high intensity in this region is consistent with the significant proportion of the cationic monomer in the compositions under study.

Signals in the δ 4.35–4.37 ppm and δ 3.66–3.69 ppm ranges are associated primarily with the methylene protons of the METAC side chain—specifically, the -COOCH_2_- and -CH_2_-N^+^ fragments, respectively.

The resonances in the δ 3.0–3.5 ppm region partially overlap. In addition to the METAC signals, methylene groups of the NVCL caprolactam ring located near the nitrogen atom may contribute to this range. Such overlap is characteristic of copolymer systems containing multiple chemically similar *N*-linked aliphatic groups.

The presence of NVCL-derived units is most clearly evident in the aliphatic region of the spectra. The signal at approximately δ 2.30 ppm is primarily associated with the methylene protons of the caprolactam ring located near the carbonyl group. Broad signals in the range of approximately δ 1.4–2.0 ppm correspond to other methylene groups of the caprolactam cycle and partially overlap with protons of the polymer backbone.

In the low-field aliphatic region (δ 0.87–1.17 ppm), broad resonances are observed, primarily attributed to the α-methyl groups of the METAC methacrylate units and other aliphatic fragments of the polymer chain.

A characteristic feature of all three spectra is significant signal broadening compared to the spectra of low-molecular-weight compounds. This behavior is typical for polymers and is associated with the restricted mobility of macromolecular chains, as well as the existence of diverse local compositional, conformational, and tactic environments for the repeating units.

The intense signal near δ 4.66 ppm is attributed to HDO present in the D_2_O solvent used and, therefore, does not originate from the structure of the copolymers under study.

In addition to the main signals associated with the polymer structure, weak resonances in the range of approximately δ 5.59–6.00 ppm are observed in all samples. These may be attributed to trace amounts of residual unsaturated compounds, including unreacted methacrylate monomer. Their significantly lower intensity compared to the signals from the saturated polymer structure is consistent with a high degree of polymerization. However, without quantitative integration analysis of these spectra, one cannot definitively claim complete monomer conversion.

As the composition shifts from 90:10 to 70:30, the aliphatic region associated with caprolactam fragments becomes more complex; this is qualitatively consistent with the increased NVCL content in the initial monomer mixture. However, a direct quantitative comparison of copolymer compositions based solely on peak heights is inappropriate, as the spectra are presented at different scales and some diagnostic signals overlap.

#### 3.1.2. FTIR Spectra of Copolymers

**Figure 3:**
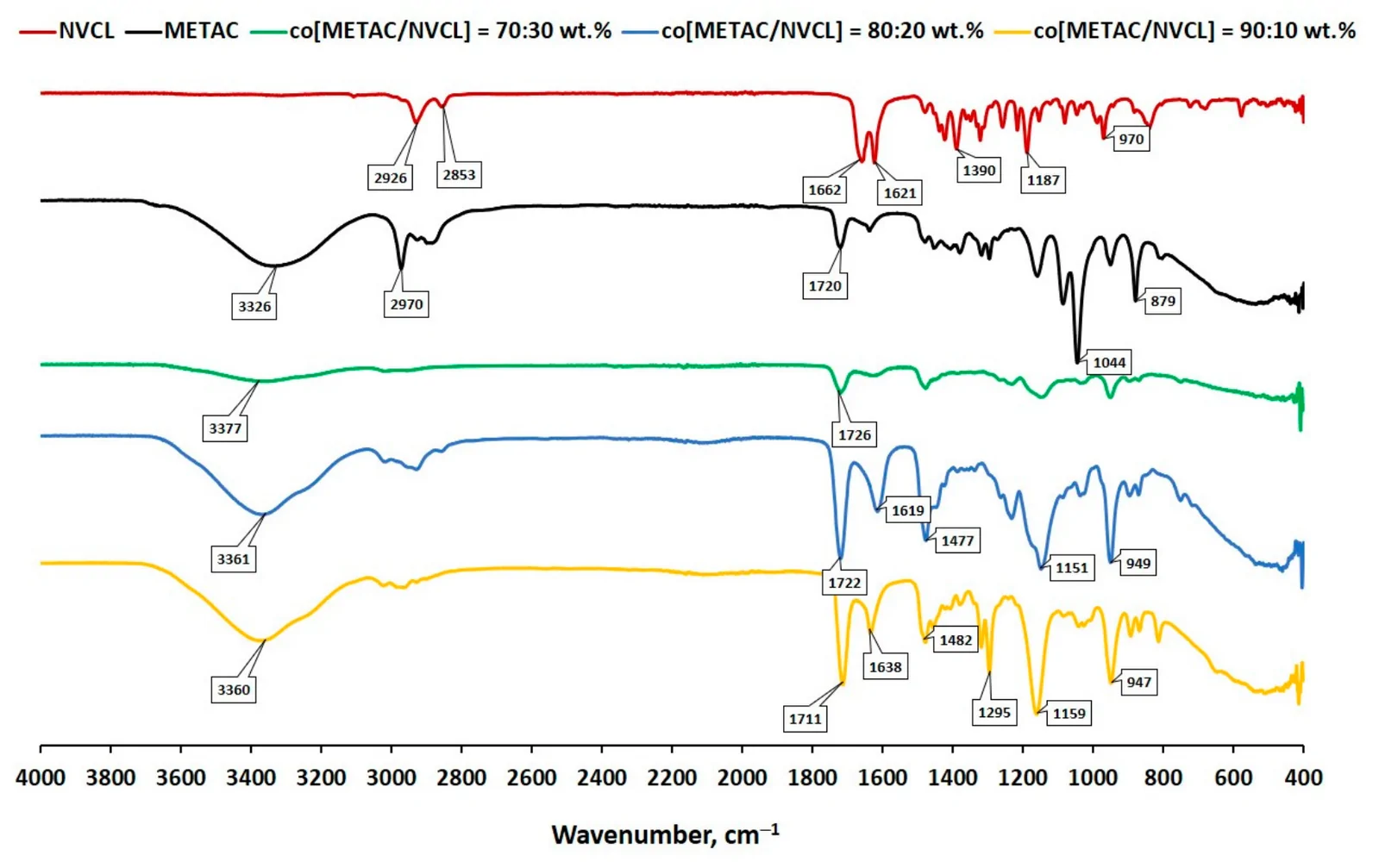
FTIR spectra of the initial monomers NVCL and METAC and the synthesized co(METAC/NVCL) copolymers with various mass ratios of the components.

The IR spectrum of *N*-vinylcaprolactam exhibits an intense band at 1659 cm^−1^, corresponding to the stretching vibrations of the lactam carbonyl group (νC=O), as well as a band at 1624 cm^−1^ [22] assigned to the stretching vibrations of the vinyl group double bond (νC=C); this confirms the monomer’s susceptibility to radical polymerization (Figure 3). The bands at 2929 and 2858 cm^−1^ correspond to the asymmetric and symmetric vibrations of C–H bonds in the methylene groups of the aliphatic ring [23]. The presence of bands in the 1330–1250 cm^−1^ region is associated with C–N bond vibrations of the amide group, characteristic of a lactam structure [24]. In the IR spectrum of [2-(methacryloyloxy)ethyl]trimethylammonium chloride, an intense band is observed at 1713 cm^−1^, corresponding to the stretching vibrations of the ester carbonyl group (νC=O), as well as a band at 1635 cm^−1^ attributed to the vibrations of the methacrylate moiety’s double bond (νC=C). The bands at 2969 cm^−1^ and 1478–1456 cm^−1^ are associated with C–H stretching and bending vibrations in alkyl and methyl groups, including the N^+^(CH_3_)_3_ group [25]. Vibrations of C–O–C and C–O bonds, characteristic of the ester group, are detected in the 1296–1161 cm^−1^ region. The presence of bands at 949–814 cm^−1^ corresponds to the out-of-plane vibrations of the vinyl group, confirming the monomeric nature of the compound. A broad band near 3370 cm^−1^ arises from O–H vibrations of sorbed moisture, a feature characteristic of quaternary ammonium salts [22].

#### 3.1.3. TGA/DSC of Copolymers

**Figure 4:**
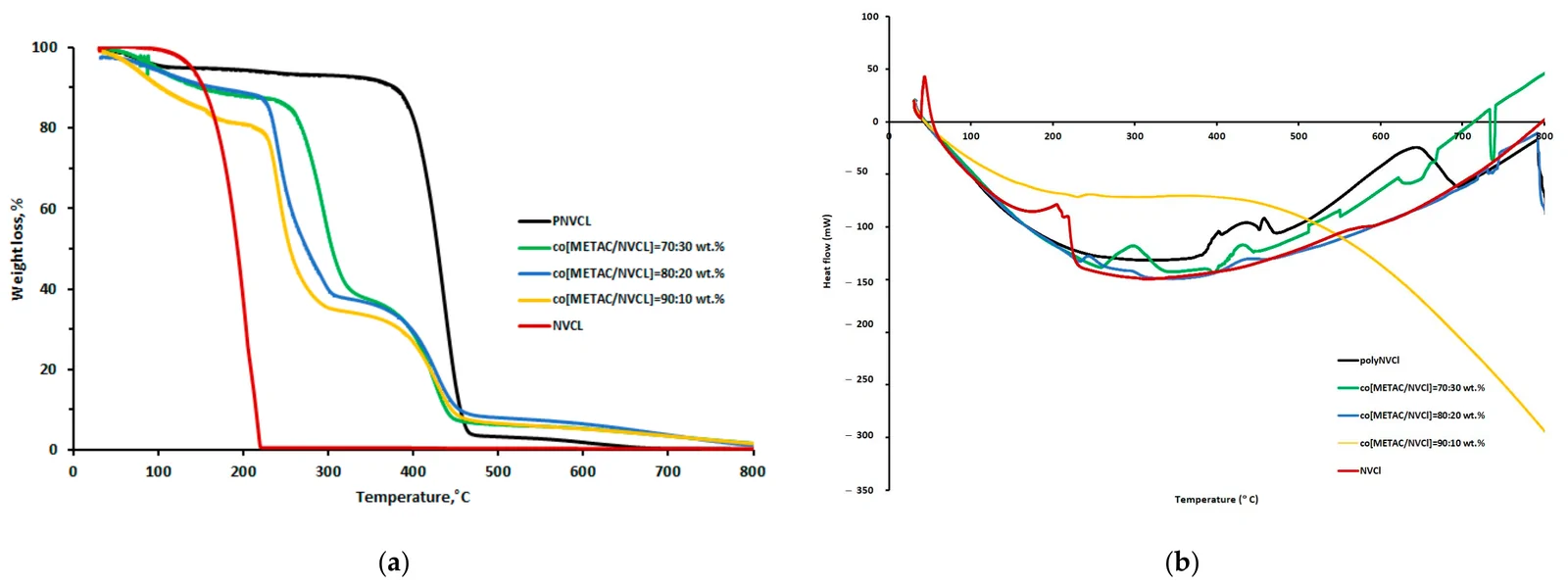
Thermal characteristics of NVCL, PNVCL, and co(METAC/NVCL) copolymers of various compositions: (**a**) TGA curves; (**b**) DSC thermograms.

The thermal behavior of the starting monomer NVCL, the homopolymer PNVCL, and the synthesized co(METAC/NVCL) copolymers was investigated using thermogravimetric analysis (TGA) and differential scanning calorimetry (DSC). The corresponding thermograms are presented in Figure 4.

According to the TGA data (Figure 4a), the NVCL monomer exhibits significantly lower thermal stability compared to PNVCL and the copolymers. A rapid mass loss for NVCL is observed in the range of approximately 150–230 °C, and the residual mass becomes minimal above 230–250 °C. This behavior is attributed to the monomer’s low molecular weight and its evaporation and/or thermal decomposition upon heating.

Polymerization of NVCL leads to a marked increase in thermal stability. For PNVCL, there is virtually no significant mass loss up to approximately 350 °C, whereas the main stage of thermal degradation occurs primarily in the 380–460 °C range. Upon completion of this stage, only a small amount of solid residue remains. Thus, the transition from the NVCL monomer to its polymeric form is accompanied by a significant shift in the main thermal decomposition region toward higher temperatures.

In contrast to PNVCL, co(METAC/NVCL) copolymers exhibit a distinct multi-stage thermal degradation pattern. The initial mass loss at temperatures up to 150 °C can be attributed primarily to the removal of physically adsorbed water and other volatile components. This effect is expected for cationic copolymers containing hydrophilic quaternary ammonium METAC groups.

The subsequent stage of mass loss occurs in the approximate range of 150–300 °C and becomes more pronounced as the METAC content increases. This stage can be linked to the onset of thermal transformation of the side groups containing quaternary ammonium moieties. The primary degradation of the polymer matrix takes place mainly between 300 °C and 450 °C, accompanied by significant mass loss resulting from the breakdown of the main macromolecular chain and functional side groups.

A comparison of the copolymers reveals a clear dependence of thermal behavior on their composition. The co(METAC/NVCL) 70:30 copolymer is characterized by a shift in the rapid mass-loss stage toward higher temperatures compared to the 80:20 and 90:10 samples. As the METAC content increases, the onset of significant thermal degradation shifts to lower temperatures. The earliest pronounced mass loss is observed for the 90:10 composition, whereas increasing the NVCL fraction enhances the stability of the copolymer matrix in this temperature range. The order of thermal stability for the investigated copolymers at the onset of rapid degradation can be qualitatively represented as co(METAC/NVCL) 70:30 > 80:20 > 90:10. This observed trend indicates that the ratio of cationic METAC units to lactam-containing NVCL units significantly influences the copolymer’s thermal decomposition mechanism.

DSC thermograms (Figure 4b) also reveal differences between NVCL, PNVCL, and copolymers of varying compositions. Thermal effects observed in the low-temperature region may be attributed to the removal of bound and adsorbed water. The copolymers exhibit additional thermal effects in the 200–300 °C range; this region coincides with the initial stage of mass loss observed in TGA data and is likely associated with transformations of the cationic functional groups of METAC.

At higher temperatures, thermal effects coincide with the range of rapid mass loss in the TGA curves, indicating the degradation of the polymer matrix. Changes in the shape and position of the DSC signals upon varying the METAC/NVCL ratio confirm that the copolymer composition significantly influences its thermal behavior.

Combined TGA and DSC analysis demonstrates that the synthesized co(METAC/NVCL) copolymers undergo multi-stage thermal degradation, involving moisture removal, transformations of cationic functional groups, and subsequent breakdown of the polymer backbone. Increasing the METAC content leads to an earlier onset of thermal degradation, whereas increasing the NVCL fraction enhances the copolymers’ stability at intermediate and high temperatures. These results further confirm that varying the METAC/NVCL ratio significantly alters the physicochemical properties of the polymer system.

### 3.2. Flotation Performance

**Figure 5:**
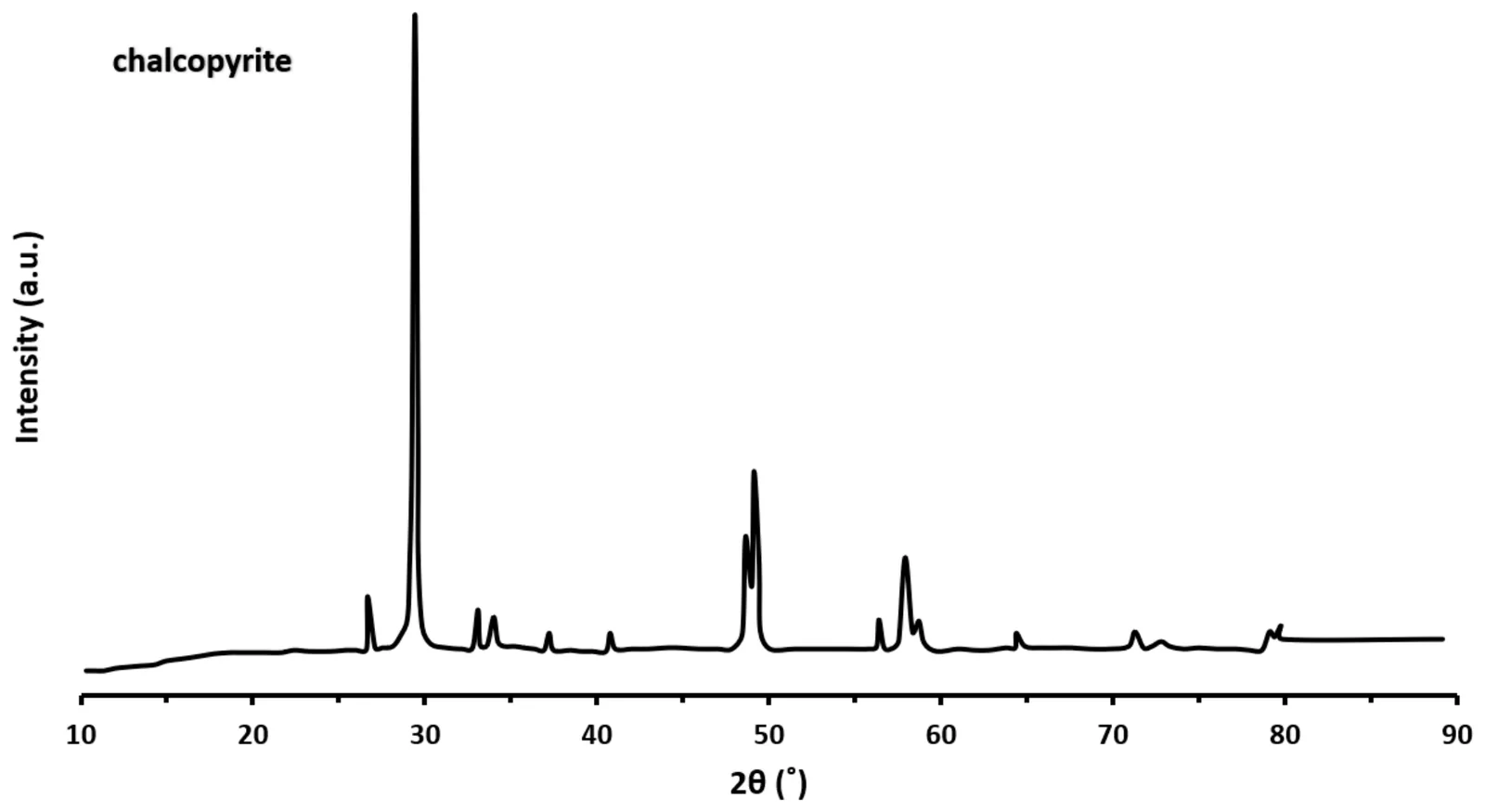
X-ray diffraction (XRD) pattern of a monomineralic chalcopyrite sample.

The phase composition of the chalcopyrite monomineral sample used in subsequent flotation experiments was investigated by X-ray diffraction (XRD). As shown in Figure 5, the diffractogram is characterized by distinct, intense reflections corresponding to the crystalline phase of chalcopyrite.

**Figure 6:**
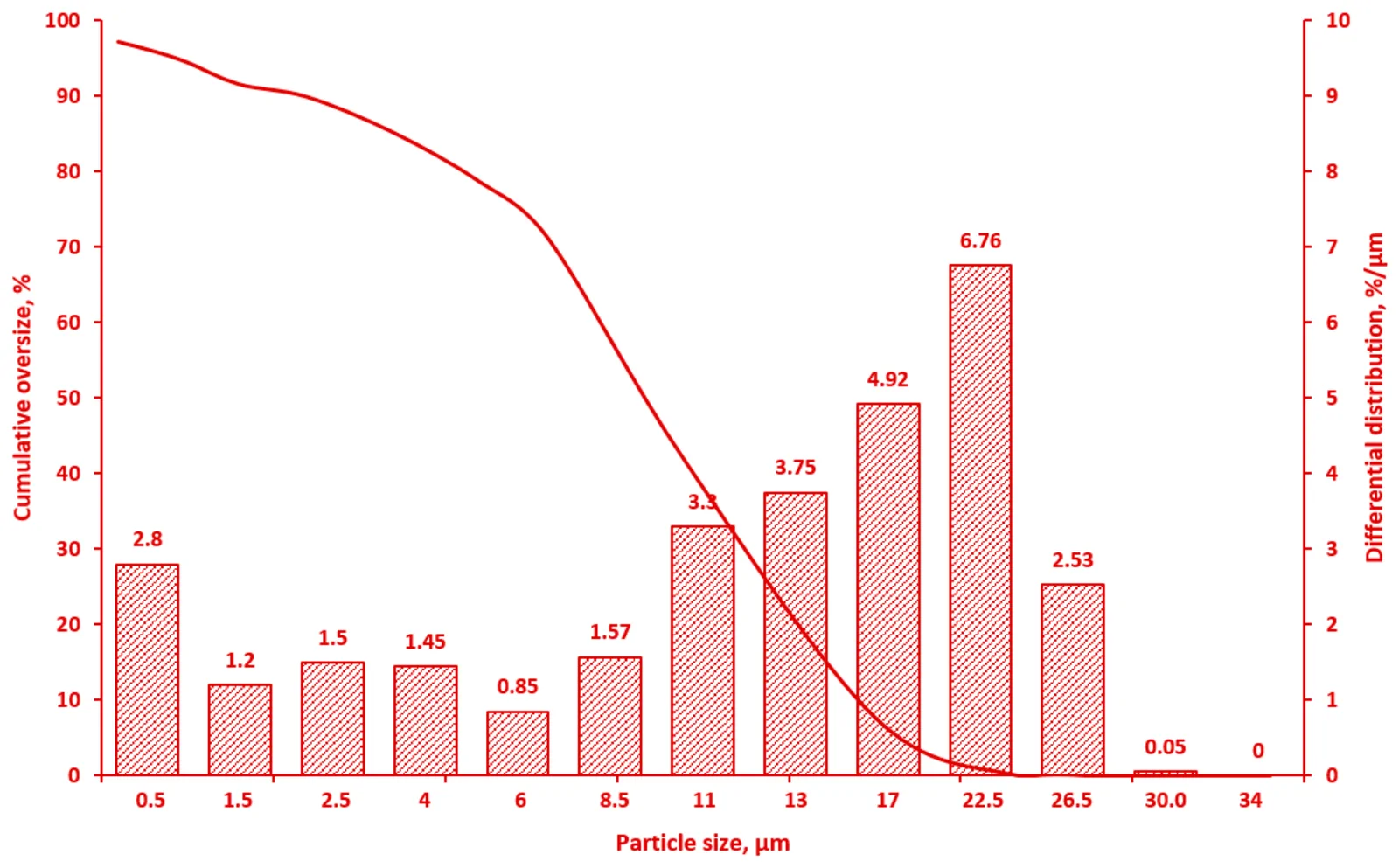
Integral and differential particle size distributions of the sample under study.

Particle size analysis was performed using the photosedimentation method to evaluate the particle size distribution of the sample. The resulting integral and differential particle size distribution curves are shown in Figure 6. The sample was found to be polydisperse, with a mean particle diameter of 18.6 µm. The largest mass fraction (63.3%) consists of particles in the 14–25 µm range, with the peak content (33.8%) observed in the 20–25 µm fraction. Fine particles smaller than 10 µm account for 14.8% of the sample, while the content of particles larger than 25 µm does not exceed 7.8%. The differential curve exhibits a distinct peak in the 20–25 µm range, indicating the predominance of this fraction in the system under study.

**Figure 7:**
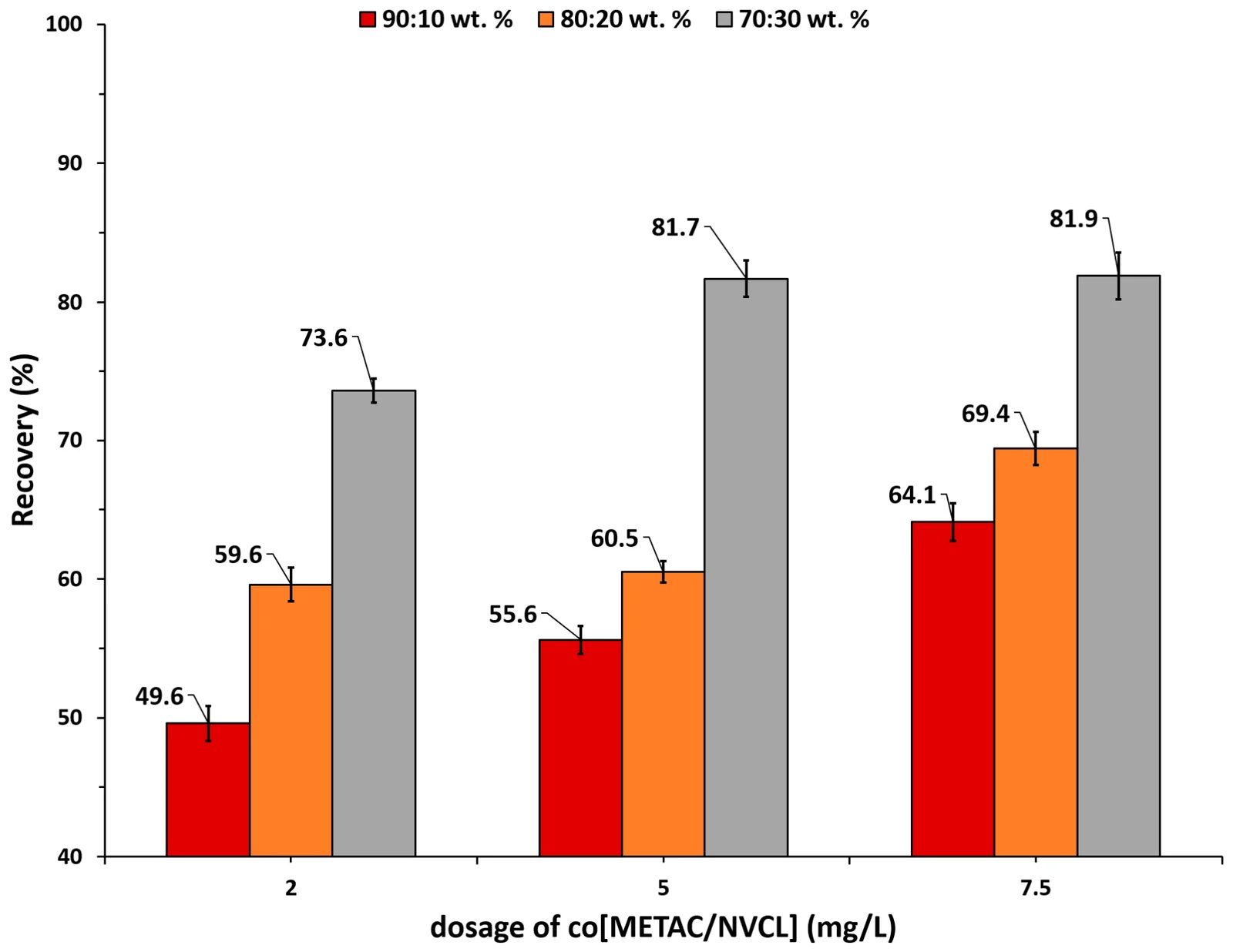
Effect of reagent concentration and METAC/NVCL feed ratio on the flotation recovery of ultrafine chalcopyrite (<30 μm) using a combined butyl xanthate co(METAC/NVCL) system at a mass ratio of 1:1, pH 8.5, and 40 °C. Concentrations refer to each reagent component.

The results show that the flotation recovery of ultrafine chalcopyrite (<30 μm) was investigated using a combined reagent system comprising butyl xanthate and co(METAC/NVCL) at a mass ratio of 1:1. The concentrations of both components were increased simultaneously, with each component present at 2.0, 5.0, or 7.5 mg/L, corresponding to total reagent concentrations of 4.0, 10.0, and 15.0 mg/L, respectively. All tests were conducted at pH 8.5 and 40 °C. At each concentration level, the formulation prepared with a higher NVCL fraction in the monomer feed gave higher chalcopyrite recovery. At 2 mg/L of each component, recoveries were 49.6%, 59.6%, and 73.6% for the METAC/NVCL feed ratios of 90:10, 80:20, and 70:30, respectively (Figure 7). At 5 mg/L of each component, the corresponding recoveries were 55.6%, 60.5%, and 81.7%, while at 7.5 mg/L, they were 64.1%, 69.4%, and 81.9%. A similar trend was observed at 5 mg/L, with recovery values of 55.6%, 60.5%, and 81.7%, respectively, and at 7.5 mg/L, with values of 64.1%, 69.4%, and 81.9%. Thus, the combined system containing the 70:30 copolymer formulation provided the highest recovery at all concentration levels tested.

The increase in recovery associated with higher NVCL content was attributed to a shift in the balance between the cationic and thermosensitive moieties of the copolymer. The quaternary ammonium groups of METAC provide a permanent positive charge to the macromolecule, potentially facilitating its anchoring onto negatively charged sites on the chalcopyrite surface at pH 8–9. Simultaneously, increasing the NVCL content enhances the contribution of thermosensitive segments to the properties of the adsorption layer. At 40 °C, reduced hydration of the NVCL-containing chain segments may strengthen hydrophobic interactions and promote a more compact conformation of the adsorbed polymer. Consequently, the particle surface may become more conducive to interaction with air bubbles. However, confirming this mechanism conclusively requires independent data regarding the phase transition temperature of the copolymers and how their surface properties change with temperature.

A particularly pronounced effect of copolymer composition is observed even at a low dosage of 2 mg/L. Shifting from a 90:10 to a 70:30 composition increased chalcopyrite recovery by 24%. At 5 mg/L, this difference was 26.1%, indicating that increasing the NVCL fraction has an impact at least as significant as simply increasing the reagent dosage. This suggests the existence of an optimal balance between electrostatic anchoring of the copolymer (driven by METAC) and interfacial interactions associated with NVCL.

Because the concentrations of butyl xanthate and the copolymer were varied together, the observed dosage dependence reflects the performance of the combined reagent system. It cannot be attributed solely to increasing the copolymer concentration. Comparisons between copolymer formulations at the same concentration level were made at equal butyl xanthate concentrations.

### 3.3. The Effect of Temperature on the Flotation Separation of Chalcopyrite and Quartz

To assess the selectivity of a combined reagent system based on the cationic copolymer Co(METAC/NVCL) and butyl xanthate, flotation tests were carried out on a synthetic mixture of chalcopyrite and quartz at temperatures of 20, 30, 40 and 50 °C. T-80 was used as the frother.

It was found that increasing the temperature from 20 to 40 °C led to an increase in copper recovery from 64.59 to 77.68% whilst simultaneously reducing SiO_2_ recovery from 7.61 to 5.78%. At the same time, the copper content in the concentrate increased from 27.43 to 30.90%, whilst the SiO_2_ content decreased from 10.06 to 7.11%.

A further increase in temperature to 50 °C was accompanied by a slight increase in copper recovery to 78.01%; however, the copper content in the concentrate fell to 30.22 per cent, whilst SiO_2_ recovery rose to 6.10% (Table 2).

Within the temperature range studied, the most favourable combination of flotation separation parameters for chalcopyrite and quartz was found at 40 °C. The results obtained demonstrate the possibility of preferential recovery of chalcopyrite from an artificial mineral mixture using a combined reagent system of Co(METAC/NVCL) and butyl xanthate in the presence of the T-80 frother.

When butyl xanthate was used on its own, an increase in temperature from 20 to 40 °C was accompanied by an increase in copper recovery from 60.45 to 72.06%. At the same time, SiO_2_ recovery decreased from 7.68 to 6.88% (Table 3). The combined use of butyl xanthate and the co(METAC/NVCL) copolymer resulted in higher copper recovery rates across the entire temperature range studied. The most pronounced difference between the reagent regimes was observed at 40 °C. Under these conditions, copper recovery increased from 72.06 to 77.68%, whilst SiO_2_ recovery decreased from 6.88 to 5.78%. At the same time, the copper content in the concentrate rose from 29.09 to 30.90%. Upon a further increase in temperature to 50 °C, copper recovery using the combined reagent system reached 78.01%; however, this was accompanied by a slight increase in SiO_2_ recovery to 6.10% and a decrease in the copper content of the concentrate to 30.22%.

### 3.4. Effect of co(METAC/NVCL) on the Wettability of the Chalcopyrite Surface

**Figure 8:**
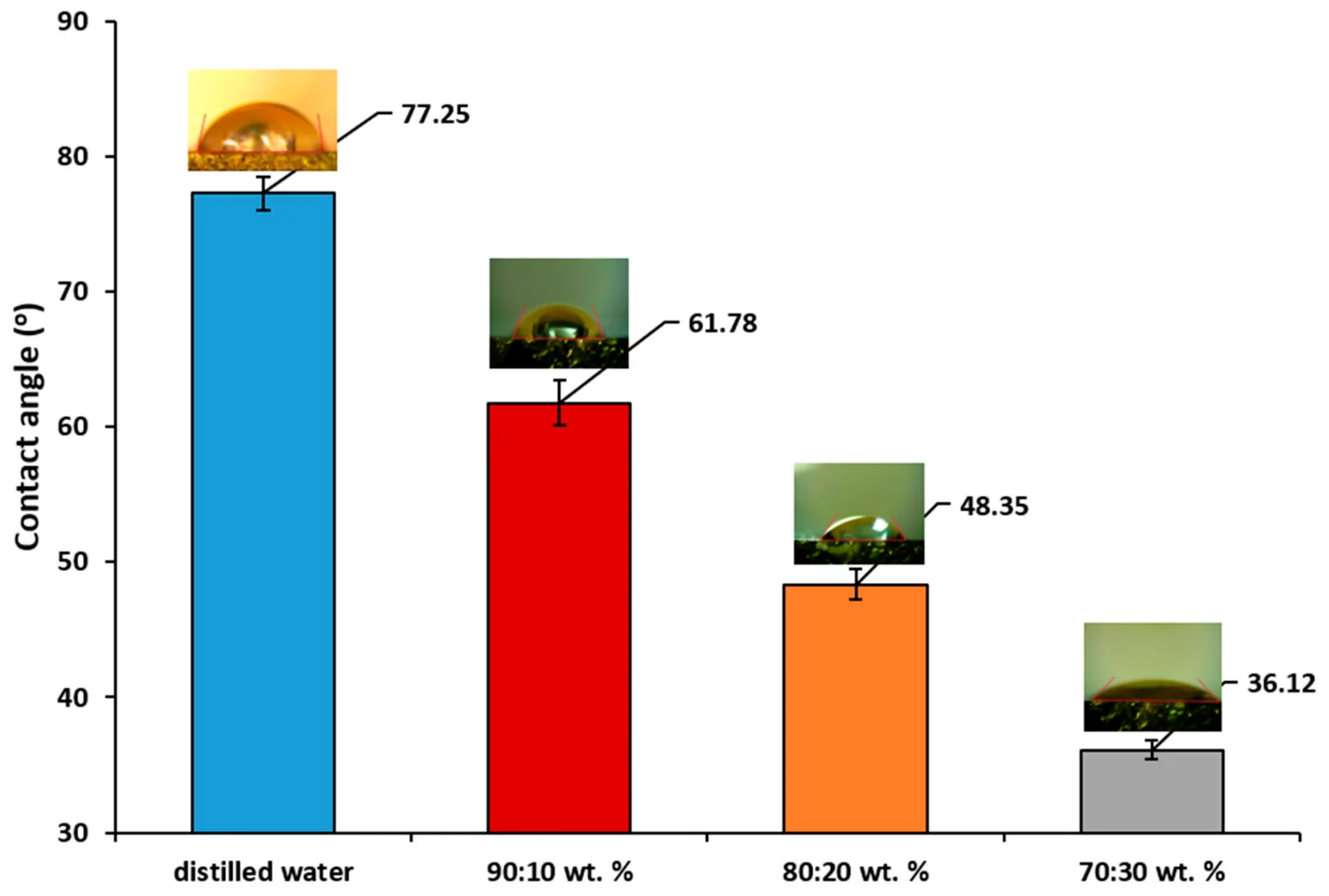
Water contact angles on a xanthate-modified chalcopyrite surface using distilled water and co(METAC/NVCL) solutions of varying composition (xanthate 5 mg/L; co(METAC/NVCL) 5 mg/L).

Contact angle measurements were performed to evaluate the effect of co(METAC/NVCL) copolymers on the wettability of a previously hydrophobized chalcopyrite surface. The polished chalcopyrite surface was pre-conditioned with a xanthate solution (concentration 5 mg/L), after which droplets of co(METAC/NVCL) copolymer solutions of various compositions (at a concentration of 5 mg/L) were applied to it. Distilled water was used as the control liquid. The obtained contact angle values are presented in Figure 8.

As shown in Figure 8, the contact angle for the control system was 77.25°. When using the co(METAC/NVCL) 90:10 solution, the value decreased to 61.78°; for the 80:20 composition, it dropped to 48.35°; and the minimum value of 36.12° was observed for co(METAC/NVCL) 70:30.

The observed trend may be attributed to changes in the interfacial properties of the copolymer solutions as the METAC/NVCL ratio is varied. The presence of a polar lactam group and non-polar hydrocarbon fragments in the NVCL structure imparts an amphiphilic character to the polymer. As noted in [11], the interaction of *N*-vinylcaprolactam units with the aqueous medium plays a significant role in determining the physicochemical properties of the resulting polymer systems. Consequently, increasing the NVCL content in the copolymers under study has the potential to alter phase-boundary interactions and the spreading behavior of the polymer solutions on the prepared mineral surface.

These results are of interest when compared with previously published studies on the effect of polymer reagents on the surface properties of sulfide minerals. Research in [26] established that polyacrylamide can adsorb onto the chalcopyrite surface and reduce its hydrophobicity under xanthate flotation conditions. The authors demonstrated that the interaction between the polymer and the mineral surface can result in a depressing effect, even in the presence of a collector. This indicates that macromolecule adsorption does not necessarily enhance the mineral’s hydrophobic properties.

Meanwhile, a study [27] on the xanthate-functionalized thermosensitive copolymer PNEXMA found that it preferentially adsorbs onto chalcopyrite compared to quartz. According to the authors, the interaction of this reagent with chalcopyrite facilitated the formation of a hydrophobic surface with contact angles exceeding 60° and enabled the reversible aggregation of finely dispersed particles. Thus, the effect of polymeric reagents on the wettability of the mineral surface is determined not only by the nature of their adsorption but also by the specifics of their chemical structure and functional composition.

Similar issues regarding the interaction of polymeric reagents with sulfide minerals are addressed in [18]. The authors demonstrated that modified polyvinylcaprolactam can adsorb onto the surfaces of chalcopyrite and galena, exhibiting selective collecting properties during flotation. These results confirm the potential of using NVCL-containing polymer systems to control interfacial interactions during the beneficiation of sulfide minerals. However, the chemical structure of the modified polyvinylcaprolactam differs from that of the co(METAC/NVCL) copolymers under study, a factor that must be considered when comparing their mechanisms of action.

Particular attention should be paid to interpreting the observed decrease in the contact angle. Since measurements were conducted using droplets of copolymer solutions with varying compositions, the observed trend characterizes the wetting of the xanthate-modified surface by the specific liquids under investigation. Study [27] demonstrated that changes in the contact angle of aqueous solutions of surface-active compounds are determined by the combined influence of interfacial adsorption at the liquid–gas and solid–liquid interfaces. Despite the differences in the compounds studied, this approach must also be taken into account when interpreting the behavior of polymer solutions.

### 3.5. X-Ray Photoelectron Spectroscopy of the Chalcopyrite Surface Before and After Treatment with co(METAC/NVCL)

**Figure 9:**
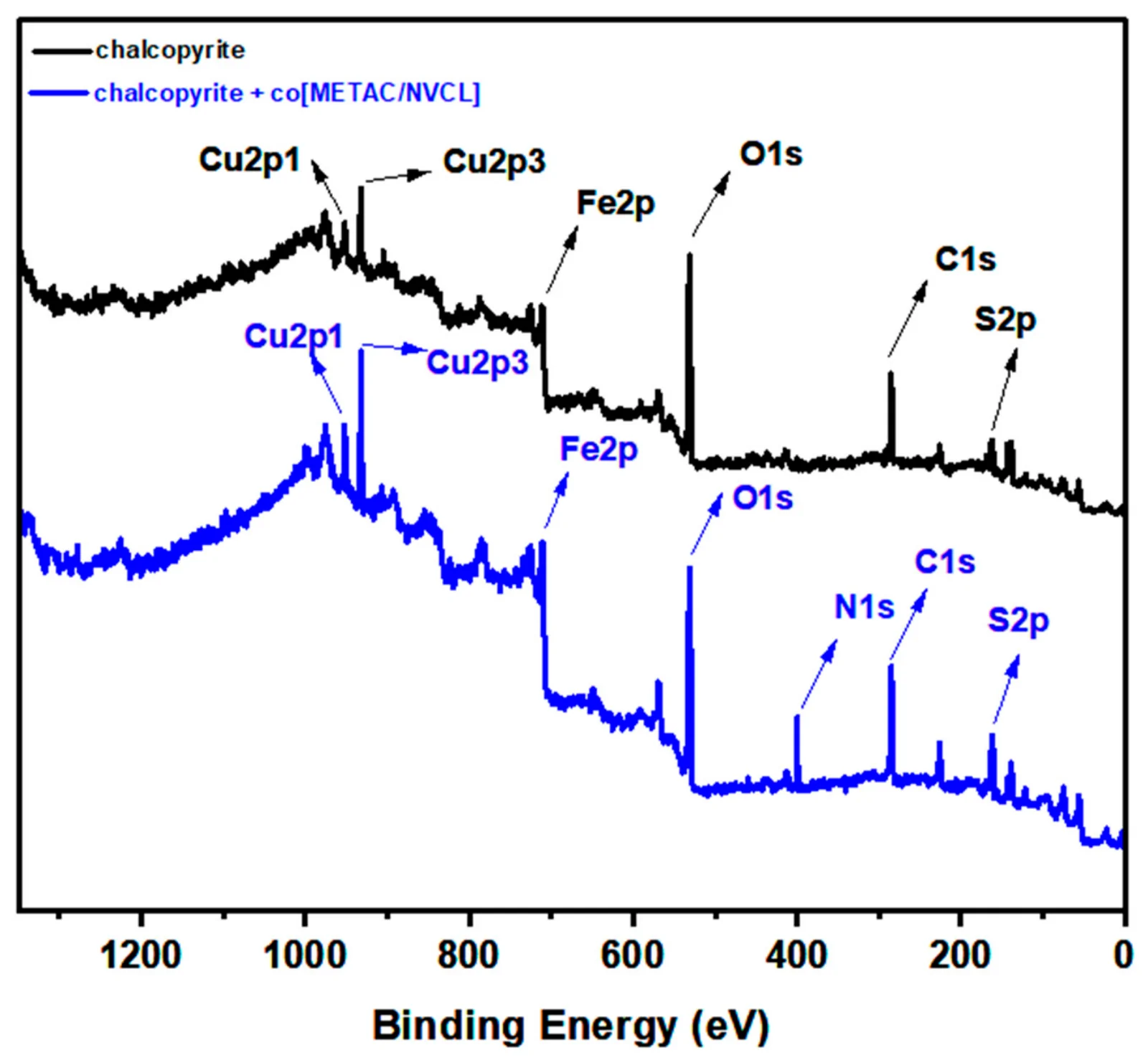
Survey XPS spectra of pristine chalcopyrite and chalcopyrite treated with the co(METAC/NVCL) copolymer.

X-ray photoelectron spectroscopy (XPS) analysis was performed to determine the nature of the interaction between the co(METAC/NVCL) copolymer and the chalcopyrite surface. Survey XPS spectra of the pristine chalcopyrite and the chalcopyrite after treatment with the copolymer are shown in Figure 9. The spectrum of the pristine mineral exhibits characteristic signals for Cu 2p, Fe 2p, S 2p, O 1s, and C 1s. Following treatment with co(METAC/NVCL), an additional N 1s signal appears in the spectrum at approximately 403 eV, a signal absent in the original sample. The appearance of a nitrogen-containing component is consistent with the chemical structure of the copolymer, which contains both NVCL lactam groups and METAC quaternary ammonium groups, and confirms the presence of a polymer layer on the chalcopyrite surface.

**Figure 10:**
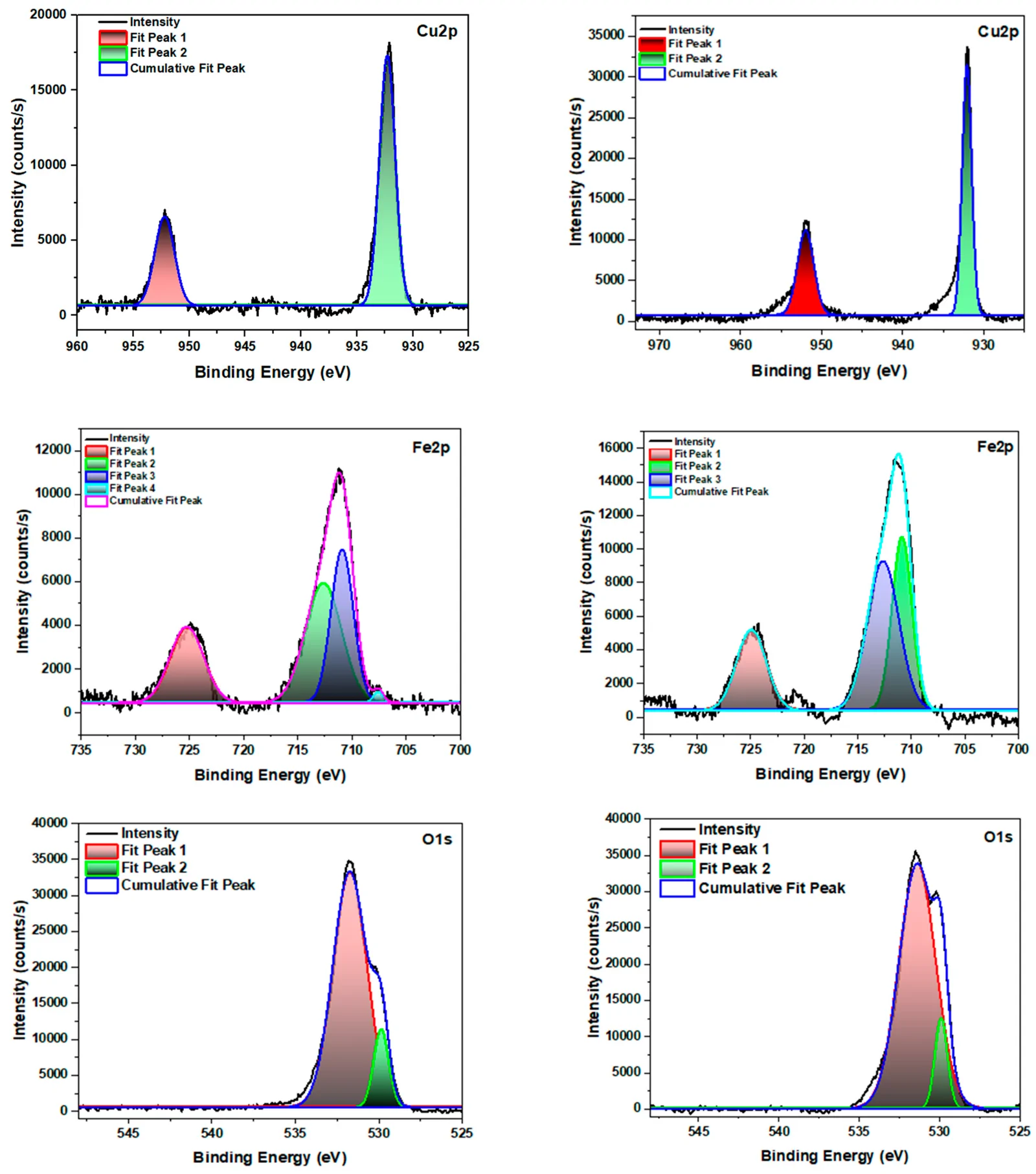

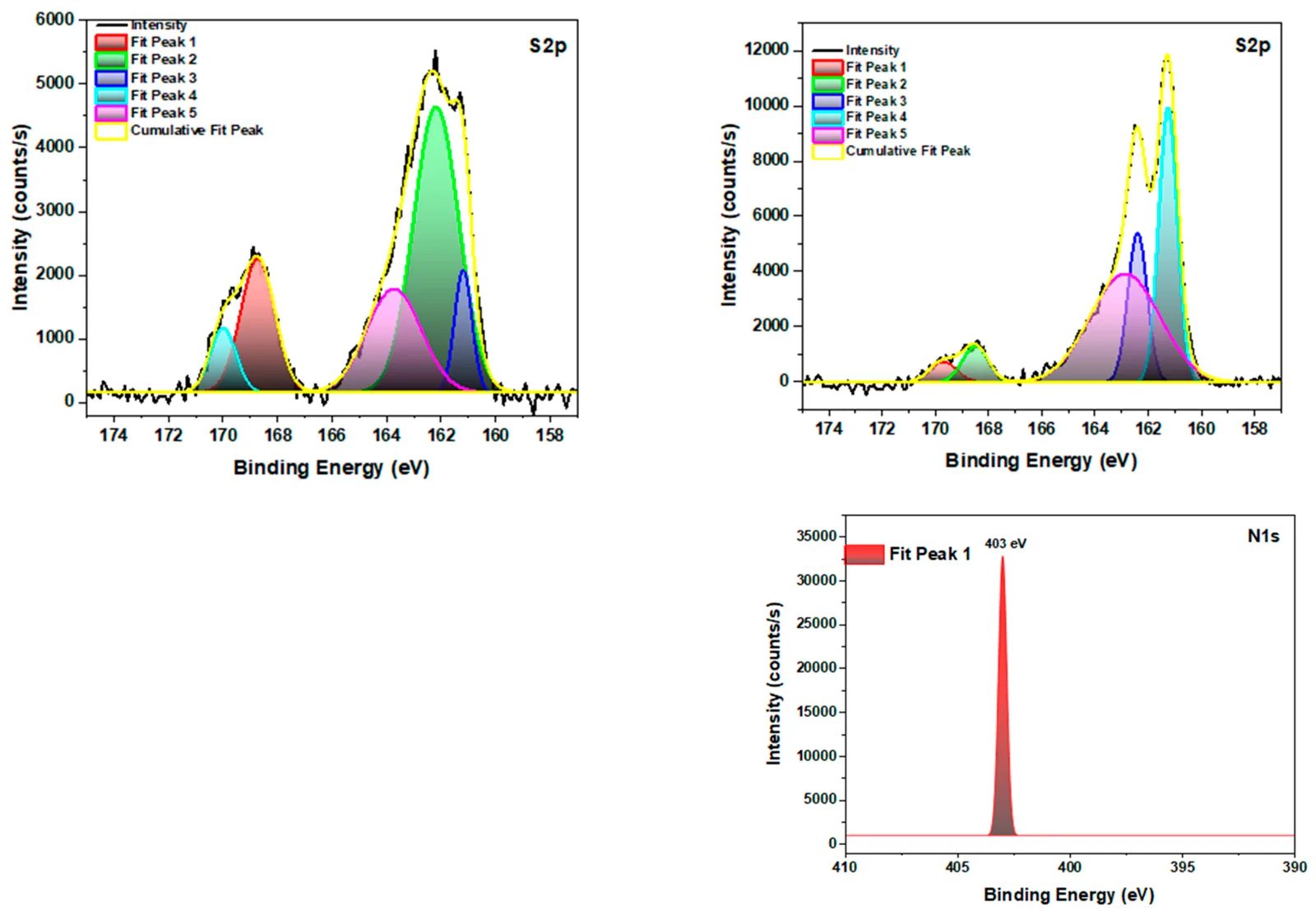
High-resolution XPS spectra and component deconvolution for Cu 2p, Fe 2p, O 1s, C 1s, S 2p, and N 1s on the chalcopyrite surface before and after co(METAC/NVCL) treatment.

To elucidate the nature of the interaction between the synthesized co(METAC/NVCL) copolymer and the chalcopyrite surface, high-resolution XPS spectra of Cu 2p, Fe 2p, O 1s, S 2p, and N 1s were analyzed. As shown in Figure 10, treatment of chalcopyrite with the copolymer results in changes to the surface composition while preserving the main spectral features of the original sulfide phase.

In the Cu 2p spectrum of pristine chalcopyrite, the main components are located at approximately 932.2–932.5 eV (Cu 2p_3_/_2_) and 952.3–952.8 eV (Cu 2p_1_/_2_). These values correspond to Cu(I)-containing sulfide forms characteristic of chalcopyrite; values around 932.5 and 952.3 eV have been reported in the literature for fresh chalcopyrite surfaces. After co(METAC/NVCL) treatment, the main maxima remain in the regions of approximately 932 and 952 eV; that is, no significant change in the chemical state of copper is observed in the obtained spectra. The absence of distinct Cu(II) satellites near 942 eV is also consistent with the predominance of Cu(I)-containing surface species. In the Fe 2p region, the spectra are characterized by a broad Fe 2p_3_/_2_ band in the approximate range of 710–713 eV and an Fe 2p_1_/_2_ component near 724–725 eV. The presence of multiple components in the 710–713 eV region indicates a heterogeneous chemical environment for iron and the possible presence of oxidized Fe-containing surface species. For chalcopyrite, components near 710–713 eV have previously been attributed to Fe(II)/Fe(III)-containing surface compounds and oxyhydroxide forms. After interaction with the copolymer, the positions of the main components change only slightly, indicating a modification of the surface layer rather than a profound transformation of the chalcopyrite crystal structure.

The O 1s spectrum can be deconvoluted into at least two components at approximately 529.8–530.0 eV and 531.5–532.0 eV. The low-energy component is characteristic of oxygen in surface metal-oxygen species, whereas the region around 531–532 eV may include contributions from hydroxyl groups and oxygen-containing functional groups in the surface layer. Similar components for the chalcopyrite surface have been reported at approximately 529.8 eV and 531.7–533 eV. For the treated sample, the high-energy O 1s contribution is particularly significant, as both copolymer units contain oxygen: NVCL contains a lactam C=O group, while METAC contains an ester moiety.

A complex multicomponent profile is observed for S 2p in both samples. The main low-energy components appear in the 161–164 eV range and correspond to various sulfide forms of sulfur characteristic of the chalcopyrite surface. Components in the higher-energy region (around 168–170 eV) can be attributed to oxidized sulfur species. Literature data for chalcopyrite indicate S^2−^ signals at approximately 161–162 eV, polysulfide forms near 163 eV, and sulfate forms at approximately 168–169 eV. The persistence of these features after treatment with co(METAC/NVCL) demonstrates that the fundamental sulfide nature of the surface is preserved.

The most significant difference is observed in the N 1s spectrum. No nitrogen signal is observed for the pristine chalcopyrite, whereas an intense component with a maximum at approximately 403.0 eV appears after treatment with the p(METAC/NVCL) copolymer. This constitutes direct spectroscopic evidence of the presence of the nitrogen-containing copolymer on the mineral surface. For PNVCL, amide nitrogen is typically observed near 399.4 eV, whereas the quaternary ammonium nitrogen of METAC appears at higher binding energies—approximately 401.7–402.8 eV—with values as high as 403–404 eV reported for some quaternary ammonium systems. Thus, the N 1s peak observed at 403 eV is most reasonably attributed to the positively charged quaternary ammonium group –N^+^(CH_3_)_3_ of the METAC unit. At 20 °C, the co(METAC/NVCL) copolymer was pre-mixed with butyl xanthate. This approach is justified by the ability of METAC-containing cationic polymers to form complexes with anionic surfactants, as reported in the literature [28,29,30]. Upon subsequent heating to 40 °C, we attribute the behaviour of the composition to the partial dehydration of the thermosensitive NVCL-containing regions and the strengthening of hydrophobic interactions. Such a temperature-dependent mechanism has been established for PNVCL [31]. At the same time, the quaternary ammonium groups of METAC are preserved and participate in interactions with xanthogenate. The combination of thermosensitivity and xanthogenate functionality provides the physicochemical basis for improved chalcopyrite flotation, which is consistent with studies of similar polymeric reagents [2,8,9,10].

## 4. Conclusions

In this study, cationic NVCL-containing co(METAC/NVCL) copolymers were synthesised via RAFT polymerisation at initial METAC/NVCL molar feed ratios of 90:10, 80:20, and 70:30. Spectroscopic characterisation using FTIR and ^1^H and ^13^C NMR supported the formation of the copolymer structure. Across the formulations prepared with increasing NVCL feed content, the weight-average molar mass (Mw) decreased from 16,230 to 8180 g/mol, while dispersity decreased from 1.46 to 1.21. Copolymer yields ranged from 72.7% to 79.2%. Thermal analysis revealed formulation-dependent differences in thermal degradation behaviour.

Single-mineral flotation tests evaluated combined reagent systems comprising butyl xanthate and co(METAC/NVCL) at a mass ratio of 1:1. At pH 8.5 and 40 °C, the system containing the 70:30 copolymer formulation provided the highest chalcopyrite recovery among the formulations tested. A recovery of 81.7% was obtained at 5 mg/L of each component. Increasing both component concentrations to 7.5 mg/L raised recovery to 81.9%, corresponding to an additional increase of only 0.2%. These results indicate a near-plateau in the measured recovery over this concentration interval. Contact-angle measurements characterised the wetting of xanthate-conditioned chalcopyrite by the copolymer solutions. XPS results, including the appearance of an N 1s signal at approximately 403 eV, were consistent with the presence of a nitrogen-containing polymer component on the treated mineral surface.

Flotation tests on a synthetic chalcopyrite–quartz mixture further demonstrated the potential of the combined reagent system. At 40 °C, the pre-mixed 70:30 copolymer formulation and butyl xanthate, each at 5 mg/L, gave a copper recovery of 77.68%, a concentrate copper grade of 30.90%, and a SiO_2_ recovery of 5.78%. Compared with butyl xanthate alone at 5 mg/L, copper recovery increased by 5.62%, while SiO_2_ recovery decreased by 1.10%. The findings support further evaluation of co(METAC/NVCL) as a component of combined xanthate-based reagent systems for chalcopyrite–quartz separation. The experiments do not establish the copolymer’s effectiveness or selectivity as an independent collector. Further research should directly investigate the solution phase behaviour of the copolymers, their interactions with butyl xanthate, and the performance of the combined system in separation from other sulphide minerals.

## Figures and Tables

**Table 1:** Characteristics of copolymers.

Sample		Ratio (wt%)	Yield (%)	M_n_ (g/mol)	M_w_ (g/mol)	D
1	co[METAC/NVCL]	90:10	79.2 ± 1.5	11,150	16,230	1.46
2		80:20	75.9 ± 2.1	9340	12,560	1.35
3		70:30	72.7 ± 1.91	6770	8180	1.21

**Table 2:** Effect of temperature on the flotation separation parameters of an artificial mixture of chalcopyrite and quartz using co(METAC/NVCL)-butyl xanthate composition.

Temperature	Product	Output		Cu, %	SiO_2,_ %	Recovery Cu, %	Recovery SiO_2,_ %
		Gram	%				
20 °C	Concentrate	3.78	37.80	27.43	10.06	64.59	7.61
	Tails	6.22	62.20	9.14	74.15	35.41	92.39
	Initial mixture	10.00	100.00	16.05	49.92	100.00	100.00
30 °C	Concentrate	3.89	38.90	29.16	8.27	69.12	6.45
	Tails	6.11	61.10	8.29	76.42	30.88	93.55
	Initial mixture	10.00	100.0	16.41	49.91	100.00	100.00
40 °C	Concentrate	4.06	40.60	30.90	7.11	77.68	5.78
	Tails	5.94	59.40	6.07	79.24	22.32	94.22
	Initial mixture	10.00	100.00	16.15	49.96	100.00	100.00
50 °C	Concentrate	4.12	41.20	30.22	7.38	78.01	6.10
	Tails	5.88	58.80	5.97	79.63	21.99	93.90
	Initial mixture	10.00	100.0	15.96	49.86	100.00	100.00

**Table 3:** Effect of temperature on the flotation separation parameters of an artificial mixture of chalcopyrite and quartz using butyl xanthate.

Temperature	Product	Output		Cu, %	SiO_2,_ %	Recovery Cu, %	Recovery SiO_2,_ %
		гр	%				
20 °C	Concentrate	3.34	33.40	27.67	11.29	60.45	7.68
	Tails	6.66	66.60	9.08	68.06	39.55	92.32
	Initial mixture	10.00	100.00	15.29	49.10	100.00	100.00
30 °C	Concentrate	3.68	36.80	28.47	9.04	67.18	6.67
	Tails	6.32	63.20	8.10	73.68	32.82	93.33
	Initial mixture	10.00	100.00	15.60	49.89	100.00	100.00
40 °C	Concentrate	3.87	38.70	29.09	8.81	72.06	6.88
	Tails	6.13	61.30	7.12	75.33	27.94	93.12
	Initial mixture	10.00	100.00	15.62	49.59	100.00	100.00
50 °C	Concentrate	3.94	39.40	29.11	8.18	73.14	6.46
	Tails	6.06	60.60	6.95	77.02	26.86	93.54
	Initial mixture	10.00	100.00	15.68	49.90	100.00	100.00

**Figure A1:**
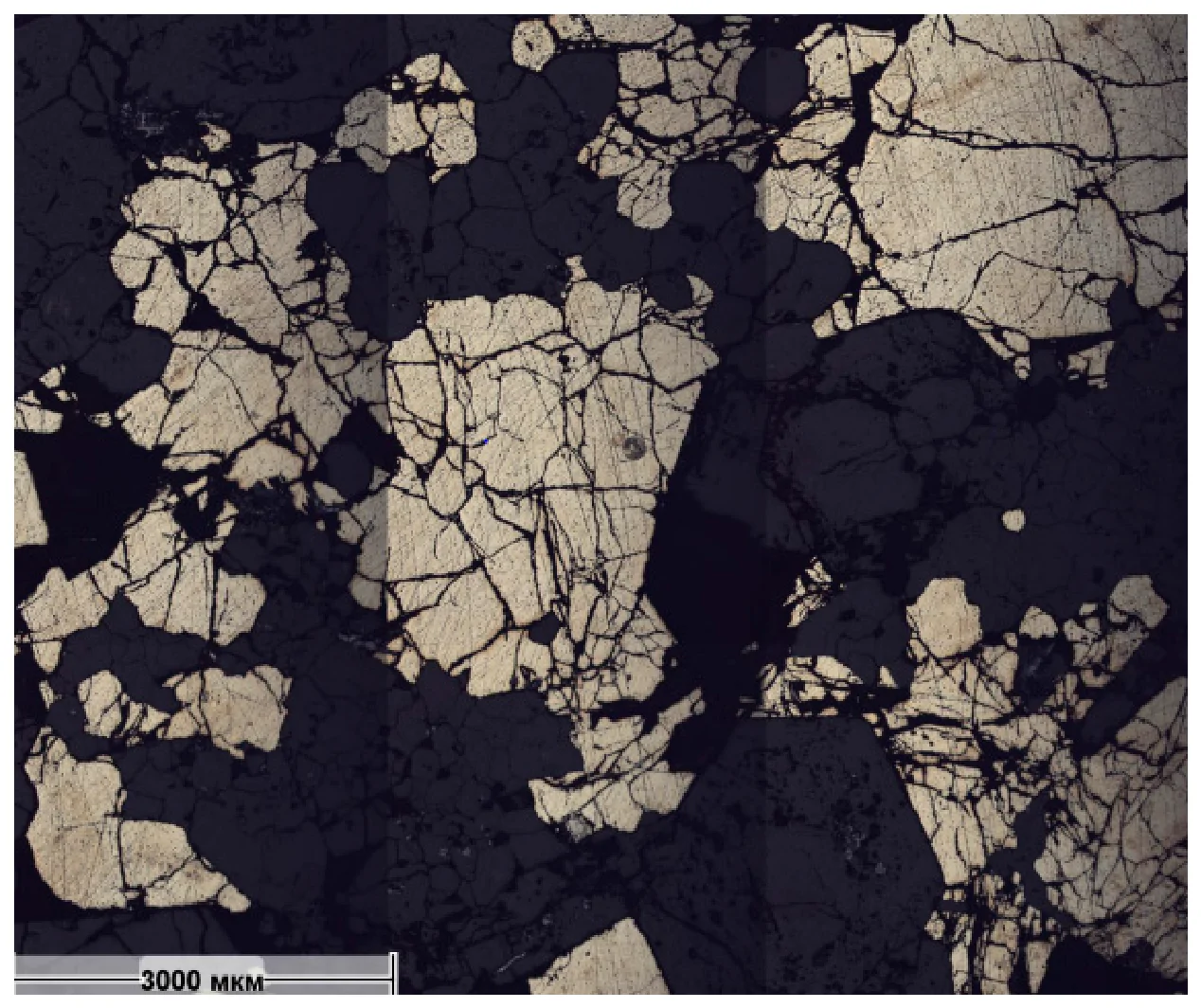
Photomicrograph of a polished section of the chalcopyrite sample under investigation.

## Data Availability

The original contributions presented in this study are included in the article. Further inquiries can be directed to the corresponding author.

## Appendix A

**Table A1:** Chemical shifts and signal assignments for ^1^H and ^13^C NMR of the co(METAC/NVCL) copolymer with a 90:10 molar ratio.

δ (ppm)	Assignment
5.998	Vinyl proton (CH_2_=C–) of the MAD-QUAT methacrylate group (residual monomer with unreacted double bond)
5.607	Second vinyl proton (CH_2_=C–) of MAD-QUAT
4.666	Residual water in D_2_O
4.367	–OCH_2_– groups of the MAD-QUAT unit (–COOCH_2_CH_2_N^+^–)
3.685	–CH_2_–N^+^ of the MAD-QUAT quaternary ammonium group
3.148	N^+^(CH_3_)_3_ of the quaternary ammonium group (main intense signal)
3.127	N^+^(CH_3_)_3_ (splitting/nonequivalence due to different microenvironments in the copolymer)
3.074	CH_2_ adjacent to the nitrogen atom of the caprolactam ring (NVCL) and overlapping N^+^(CH_3_)_3_ signal
3.036	CH_2_ of the NVCL caprolactam ring, partially overlapping with MAD-QUAT signals
2.306	CH_2_ of the α-carbonyl group of the caprolactam ring (–COCH_2_–)
1.876	Methine protons of the polymer backbone and CH_2_ of the caprolactam ring
1.773	CH_2_ of the backbone and caprolactam ring
1.601	Remaining methylene groups of the NVCL caprolactam ring
1.454	Methylene groups of the polymer backbone
1.165	Methyl group of the polymethacrylate backbone fragment (α-CH_3_)
1.009 and 0.959	Backbone methyl groups in different tacticity environments
0.881	Terminal methyl groups of the polymer chain

**Table A2:** Chemical shifts and signal assignments for ^1^H and ^13^C NMR of the co(METAC/NVCL) copolymer with an 80:20 molar ratio.

δ (ppm)	Assignment
5.998	Vinyl proton (CH_2_=C–) of the MAD-QUAT methacrylate group (residual monomer with unreacted double bond)
5.607	Second vinyl proton (CH_2_=C–) of MAD-QUAT
4.666	Residual water in D_2_O
4.367	–OCH_2_– groups of the MAD-QUAT unit (–COOCH_2_CH_2_N^+^–)
3.898	N^+^(CH_3_)_3_ quaternary ammonium MAD-QUAT
3.685	–CH_2_–N^+^ of the MAD-QUAT quaternary ammonium group
3.495	CH_2_–N^+^ and, to some extent, the CH_2_ groups of the NVCL ring
3.359	CH_2_ near the nitrogen atom in NVCL;
3.148	N^+^(CH_3_)_3_ of the quaternary ammonium group (main intense signal)
3.127	N^+^(CH_3_)_3_ (splitting/nonequivalence due to different microenvironments in the copolymer)
3.074	CH_2_ adjacent to the nitrogen atom of the caprolactam ring (NVCL) and overlapping N^+^(CH_3_)_3_ signal
3.036	CH_2_ of the NVCL caprolactam ring, partially overlapping with MAD-QUAT signals
2.306	CH_2_ of the α-carbonyl group of the caprolactam ring (–COCH_2_–)
1.876	Methine protons of the polymer backbone and CH_2_ of the caprolactam ring
1.773	CH_2_ of the backbone and caprolactam ring
1.601	Remaining methylene groups of the NVCL caprolactam ring
1.454	Methylene groups of the polymer backbone
1.165	Methyl group of the polymethacrylate backbone fragment (α-CH_3_)
1.009 and 0.959	Backbone methyl groups in different tacticity environments
0.881	Terminal methyl groups of the polymer chain

**Table A3:** Chemical shifts and signal assignments for ^1^H and ^13^C NMR of the co(METAC/NVCL) copolymer with a 70:30 molar ratio.

δ (ppm)	Assignment
5.982	Vinyl proton (CH_2_=C–) of the MAD-QUAT methacrylate group (residual monomer with unreacted double bond)
5.592	Second vinyl proton (CH_2_=C–) of MAD-QUAT
4.663	Residual water in D_2_O
4.352	–OCH_2_– groups of the MAD-QUAT unit (–COOCH_2_CH_2_N^+^–)
3.882	N^+^(CH_3_)_3_ quaternary ammonium MAD-QUAT
3.664	–CH_2_–N^+^ of the MAD-QUAT quaternary ammonium group
3.479	CH_2_–N^+^ and, in part, CH_2_ of the NVCL ring
3.337	CH_2_ near the nitrogen atom in NVCL;
3.130	N^+^(CH_3_)_3_ of the quaternary ammonium group (main intense signal)
3.057	N^+^(CH_3_)_3_ (splitting/nonequivalence due to different microenvironments in the copolymer)
3.019	CH_2_ adjacent to the nitrogen atom of the caprolactam ring (NVCL) and overlapping N^+^(CH_3_)_3_ signal
2.872	CH_2_ of the NVCL caprolactam ring, which partially overlaps with the MAD-QUAT signals
2.743	CH_2_ of the caprolactam ring. Additional chemical environment.
2.303	CH_2_ of the α-carbonyl group of the caprolactam ring (–COCH_2_–)
2.025	the CH_2_ group of the caprolactam ring
1.876	Methine protons of the polymer backbone and CH_2_ of the caprolactam ring
1.759	CH_2_ of the backbone and caprolactam ring
1.597	Remaining methylene groups of the NVCL caprolactam ring
1.427	Methylene groups of the polymer backbone
1.259	CH_2_ in the main chain
1.153	Methyl group of the polymethacrylate backbone fragment (α-CH_3_)
1.092	CH_3_ in the initiator or end groups
0.994	CH_3_ terminal groups
0.944	CH_3_ from AIBA
0.868	Terminal methyl groups on the polymer chain

## References

[1] Bednarek P.S., Zawala J., Kowalczuk P.B. (2025). Polymer-based collectors in flotation: A review. Adv. Colloid Interface Sci..

[2] Ng W.S., Connal L.A., Forbes E., Franks G.V. (2016). Xanthate-functional temperature-responsive polymers as selective flocculants and collectors for fines recovery. Miner. Eng..

[3] Liu X., Du Z., Sun C., Zhang N. (2025). A review on the electrochemical analysis of sulfide minerals—Pyrite, chalcopyrite, and galena. Green Smart Min. Eng..

[4] Chen J., Zhang Y. (2025). Large system study of chalcopyrite and pyrite flotation surfaces based on SCC-DFTB parameterization method. Int. J. Min. Sci. Technol..

[5] Wang Z., Shi Q., Zhang G., Zhu Y., Li B. (2025). Effect of pyrite content on chalcopyrite flotation under different regrinding conditions. Int. J. Miner. Metall. Mater..

[6] Wang X., Wen S., Chen S., Zhang S., Feng F., Han G., Feng Q. (2026). Tetramethylthiourea as a highly selective collector for the flotation separation of chalcopyrite from pyrite under low-alkalinity conditions. Green Smart Min. Eng..

[7] Xie Y., Yin W., Liu Q., Wang D., Sun W. (2025). Reverse floc-flotation of talc from chalcopyrite by using polyvinyl acetate as a flocculant: Adsorption and bubble capture studies. Int. J. Min. Sci. Technol..

[8] Ng W.S., Forbes E., Franks G.V., Connal L.A. (2016). Xanthate-Functional Temperature-Responsive Polymers: Effect on Lower Critical Solution Temperature Behavior and Affinity toward Sulfide Surfaces. Langmuir.

[9] Ng W.S., Cooper L., Connal L.A., Forbes E., Jameson G.J., Franks G.V. (2018). Tuneable collector/depressant behaviour of xanthate-functional temperature-responsive polymers in the flotation of copper sulfide: Effect of shear and temperature. Miner. Eng..

[10] Ng W.S., Sonsie R., Forbes E., Franks G.V. (2015). Flocculation/flotation of hematite fines with anionic temperature-responsive polymer acting as a selective flocculant and collector. Miner. Eng..

[11] Cortez-Lemus N.A., Licea-Claveríe A. (2016). Poly(*N*-vinylcaprolactam), a comprehensive review on a thermoresponsive polymer becoming popular. Prog. Polym. Sci..

[12] Ponce-Vargas S.M., Cortez-Lemus N.A., Licea-Claveríe A. (2013). Preparation of Poly(*N*-Vinylcaprolactam) (NVCL) and Statistical Copolymers of NVCL with Variable Cloud Point Temperature by Using a Trithiocarbonate RAFT Agent. Macromol. Symp..

[13] Shatalov G.V., Verezhnikov V.N., Plaksitskaya T.V., Kuznetsov V.A., Poyarkova T.N., Yan’shina A.V. (2006). Synthesis of *N*,*N*-dimethylaminoethyl methacrylate copolymers with *N*-vinylcaprolactam and their complexing and flocculating behavior. Polym. Sci. Ser. A.

[14] Wei M., Ban B., Li J., Sun J., Li F., Jiang X., Chen J. (2020). Flotation behavior, collector adsorption mechanism of quartz and feldspar-quartz systems using PEA as a novel green collector. Silicon.

[15] Jiang X., Shi J., Chen C., Song W., Ban B., Li J., Wang A., Chen J. (2021). Flotation mechanism and application of PEA with different chain lengths in quartz flotation. Chem. Eng. Sci..

[16] Murga R., Rodriguez C., Amalraj J., Vega-Garcia D., Gutierrez L., Uribe L. (2022). Use of polystyrene nanoparticles as collectors in the flotation of chalcopyrite. Polymers.

[17] Matveeva T.N., Getman V.V., Karkeshkina A.Y. (2021). Study of the use of polyvinylcaprolactam in the reagent mode of gold flotation. Tsvetnye Met..

[18] Matveeva T.N., Getman V.V., Karkeshkina A.Y. (2023). Investigation of the interaction of modified polyvinyl caprolactam with the surface of minerals included in the composition of polycomponent ores. Tsvetnye Met..

[19] Matveeva T.N., Getman V.V., Karkeshkina A.Y. (2024). Effect of Modified Polyvinyl Caprolactam on Gold Recovery from Fine Slime in Gold Ore Flotation. J. Min. Sci..

[20] Ahmadi R., Khodadadi Darban A., Abdollahy M., Fan M. (2014). Nano-microbubble flotation of fine and ultrafine chalcopyrite particles. Int. J. Min. Sci. Technol..

[21] Chen W., Liu Z., Tian Y., Long Y., Yang J. (2026). Cellulose-derived quaternary ammonium salt depressant: Preparation, and clean flotation separation of molybdenite from chalcopyrite. Miner. Eng..

[22] Halligan E., Tie B.S.H., Colbert D.M., Alsaadi M., Zhuo S., Keane G., Geever L.M. (2023). Synthesis and Characterisation of Hydrogels Based on Poly (*N*-Vinylcaprolactam) with Diethylene Glycol Diacrylate. Gels.

[23] Lee B., Jiao A., Yu S., You J.B., Kim D.-H., Im S.G. (2013). Initiated chemical vapor deposition of thermoresponsive poly(*N*-vinylcaprolactam) thin films for cell sheet engineering. Acta Biomater..

[24] Gola A., Niżniowska A., Musiał W. (2019). The Influence of Initiator Concentration on Selected Properties on Poly-*N*-Vinylcaprolactam Nanoparticles. Nanomaterials.

[25] Zhang Y., Luo Y., Wang L., Ng P.F., Hu H., Chen F., Huang Q., Zheng Z. (2022). Destructive-treatment-free rapid polymer-assisted metal deposition for versatile electronic textiles. ACS Appl. Mater. Interfaces.

[26] Huang P., Wang L., Liu Q. (2014). Depressant function of high molecular weight polyacrylamide in the xanthate flotation of chalcopyrite and galena. Int. J. Miner. Process..

[27] Staniscia F., Guzman H.V., Kanduč M. (2022). Tuning Contact Angles of Aqueous Droplets on Hydrophilic and Hydrophobic Surfaces by Surfactants. J. Phys. Chem. B.

[28] Bastardo L.A., Iruthayaraj J., Lundin M., Dedinaite A., Vareikis A., Makuška R., van der Wal A., Furó I., Garamus V.M., Claesson P.M. (2007). Soluble complexes in aqueous mixtures of low charge density comb polyelectrolyte and oppositely charged surfactant probed by scattering and NMR. J. Colloid Interface Sci..

[29] Khutoryanskiy V.V., Nurkeeva Z.S., Mun G.A., Sergaziyev A.D., Kadlubowski S., Fefelova N.A., Baizhumanova T., Rosiak J.M. (2004). Temperature-responsive linear polyelectrolytes and hydrogels based on [2-(methacryloyloxy)ethyl] trimethylammonium chloride and *N*-isopropylacrylamide and their complex formation with potassium hexacyanoferrates (II, III). J. Polym. Sci. B Polym. Phys..

[30] Mun G.A., Khutoryanskiy V.V., Nurkeeva Z.S., Sergaziyev A.D., Fefelova N.A., Rosiak J.M. (2004). Polyelectrolyte complexes of linear copolymers and hydrogels based on 2-[(methacryloyloxy)ethyl]trimethylammonium chloride and *N*-isopropylacrylamide. J. Polym. Sci. B Polym. Phys..

[31] Sun X., Qian X. (2019). Atomistic molecular dynamics simulations of the lower critical solution temperature transition of poly(*N*-vinylcaprolactam) in aqueous solutions. J. Phys. Chem. B.

